# The UDPase ENTPD5 regulates ER stress-associated renal injury by mediating protein N-glycosylation

**DOI:** 10.1038/s41419-023-05685-4

**Published:** 2023-02-27

**Authors:** Lifen Xu, Yuxia Zhou, Guifang Wang, Li Bo, Bangming Jin, Lujun Dai, Qinli Lu, Xueni Cai, Laying Hu, Lu Liu, Yixuan Wu, Xuebing Chang, Yali Huang, Lingyu Song, Tian Zhang, Yuanyuan Wang, Ying Xiao, Fan Zhang, Lingling Liu, Mingjun Shi, Tuanlao Wang, Bing Guo

**Affiliations:** 1 International Scientific and Technological Cooperation Base of Pathogenesis and Drug Research on Common Major Diseases, Guizhou Medical University, 550025 Guiyang, China; 2 Guizhou Provincial Key Laboratory of Pathogenesis and Drug Research on Common Chronic Diseases, Guizhou Medical University, 550025 Guiyang, China; 3 Department of Pathophysiology, Guizhou Medical University, 550025 Guiyang, China; 4 Department of Pathology, Affiliated Hospital of Guizhou Medical University, 550004 Guiyang, China; 5 Department of Physiology, Guizhou Medical University, 550025 Guiyang, China; 6 School of Pharmaceutical Sciences, State Key Laboratory of Cellular Stress Biology, Fujian Provincial Key Laboratory of Innovative Drug Target Research, Xiamen University, 361005 Xiamen, China

**Keywords:** Kidney, Kidney diseases

## Abstract

Impaired protein N-glycosylation leads to the endoplasmic reticulum (ER) stress, which triggers adaptive survival or maladaptive apoptosis in renal tubules in diabetic kidney disease (DKD). Therapeutic strategies targeting ER stress are promising for the treatment of DKD. Here, we report a previously unappreciated role played by ENTPD5 in alleviating renal injury by mediating ER stress. We found that ENTPD5 was highly expressed in normal renal tubules; however, ENTPD5 was dynamically expressed in the kidney and closely related to pathological DKD progression in both human patients and mouse models. Overexpression of ENTPD5 relieved ER stress in renal tubular cells, leading to compensatory cell proliferation that resulted in hypertrophy, while ENTPD5 knockdown aggravated ER stress to induce cell apoptosis, leading to renal tubular atrophy and interstitial fibrosis. Mechanistically, ENTPD5-regulated N-glycosylation of proteins in the ER to promote cell proliferation in the early stage of DKD, and continuous hyperglycemia activated the hexosamine biosynthesis pathway (HBP) to increase the level of UDP-GlcNAc, which driving a feedback mechanism that inhibited transcription factor SP1 activity to downregulate ENTPD5 expression in the late stage of DKD. This study was the first to demonstrate that ENTPD5 regulated renal tubule cell numbers through adaptive proliferation or apoptosis in the kidney by modulating the protein N-glycosylation rate in the ER, suggesting that ENTPD5 drives cell fate in response to metabolic stress and is a potential therapeutic target for renal diseases.

## Introduction

Renal hypertrophy is a major morphological change in the early stage of diabetic kidney disease (DKD) and is characterized by expanded and enlarged glomeruli that contain more tubular cells than those in healthy kidneys [1–3]. Although renal hypertrophy is initially a compensatory or adaptive change, it eventually contributes to renal maladaptation, resulting in apoptosis, tubular atrophy and renal fibrosis [4, 5]. Therefore, it is important to examine the mechanism of this alteration by focusing on key components involved in pathological DKD progression and searching for new therapeutic strategies to revitalize renal tubules and increase their integrity.

Glucose is a fuel source for energy metabolism and a regulatory signal indicating protein modifications, such as glycosylation [6, 7]. Aberrant glucose metabolism in DKD may lead to abnormal glycosylation, which drives DKD progression [8, 9]. N-glycosylation (N-acetylglucosamine, GlcNAc) crucially regulates the maturation and quality control of protein synthesis and controls receptor translocation to the plasma membrane, where it promotes cell growth and proliferation [7]. Impaired glycosylation often results in the disruption of protein maturation and incorrect protein folding, inducing the unfolded protein response (UPR) in the endoplasmic reticulum (ER) [10, 11]. UPR-mediated ER stress can trigger adaptive survival responses [12] or cell death [13].

Ectonucleoside triphosphate diphosphohydrolase 5 (ENTPD5), a nucleotide hydrolase located in the ER, hydrolyzes UDP to UMP, is mediated by UGGT, and promotes the correct folding of N-glycoproteins in the ER [14]. ENTPD5 promotes tumor proliferation through ATP consumption and favors aerobic glycolysis [14, 15]. The transcription factor SP1 promotes mutp53 binding to the ENTPD5 promoter, which can accelerate tumor progression and metastasis [16]. In addition, ENTPD5 participates in HRD1-mediated (an ER-associated ubiquitin ligase) regulation of liver metabolism [17]. However, the role of ENTPD5 in the kidney has not been examined.

We found that ENTPD5 was highly expressed in the proximal renal tubules but not in the glomerulus, which prompted us to examine the role of ENTPD5 in the kidney. Further examination revealed that ENTPD5 expression in the kidney was closely related to pathological DKD progression in human patients and mouse models. Herein, we present data demonstrating that ENTPD5 overexpression effectively alleviates kidney failure and in contrast, that ENTPD5 downregulation exacerbates kidney failure. Our results suggest that ENTPD5 prevents renal tubular cells from undergoing adaptive proliferation or apoptosis by modulating protein N-glycosylation in the ER.

## Results

### Dynamic expression of ENTPD5 in the kidneys of DKD patients and diabetic mice

**Fig. 1:**
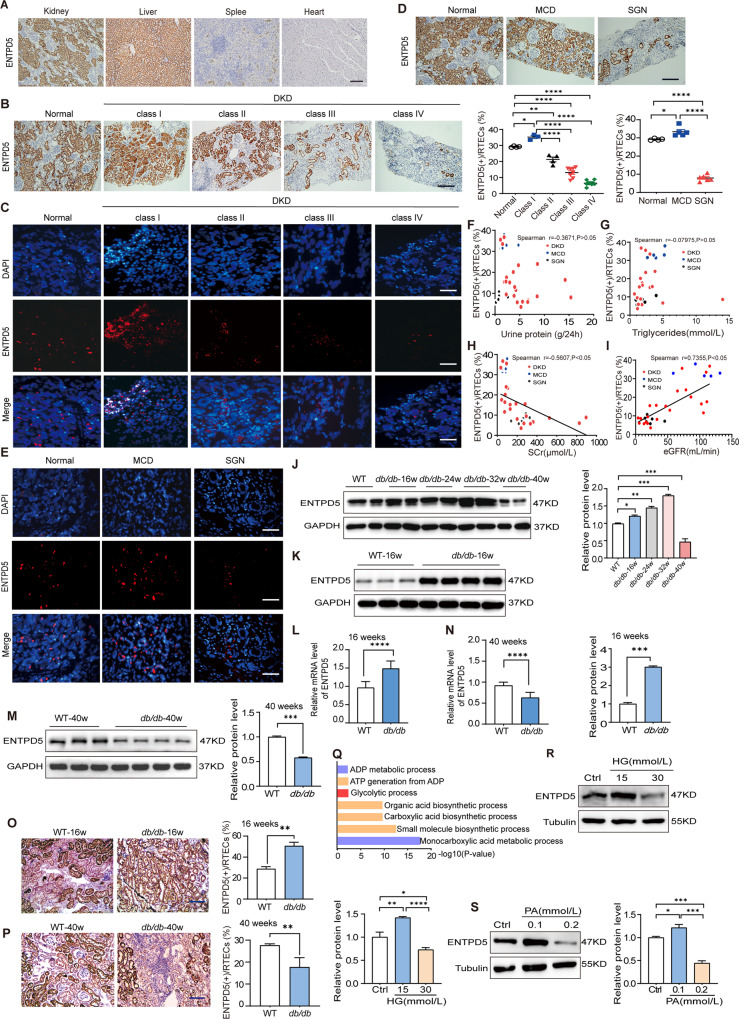
Dynamic expression of ENTPD5 in the kidneys of DKD patients and diabetic mice. **A** Representative IHC images of ENTPD5 expression in healthy adult kidney, liver, spleen, and heart tissues. Scale bar, black 200 μm. **B** Representative IHC images and quantification of proximal tubular ENTPD5 expression in renal tissues from DKD patients: Normal human kidney tissue (*n* = 4), subjects with mild lesion type (class I, *n* = 3), thylakoid hyperplasia type (class II, *n* = 4), tuberous sclerosis type (class III, *n* = 8), and advanced glomerulosclerosis (class IV, *n* = 7). Scale bar, black 200 μm. **C** Representative fluorescence images of ENTPD5 mRNA levels detected by FISH assay in the kidneys of normal human and different histological grades of DKD patients. Scale bar, white 50 μm. **D** Representative IHC images and quantification of ENTPD5 expression in renal tissues from patients with MCD and SGN, normal human kidney tissue (*n* = 4), MCD (*n* = 5), SGN (*n* = 6). Scale bar, black 200 μm. **E** Representative fluorescence images of ENTPD5 mRNA levels detected by FISH assay in the kidneys of normal human, MCD, and SGN patients. Scale bar: white 50 μm. **F**–**I** Correlation of ENTPD5 expression in renal tubules with 24-h urine protein quantification (*n* = 33) (**F**), serum triglycerides (*n* = 33) (**G**), serum creatinine (SCr, *n* = 33) (**H**), and eGFR (*n* = 33) (**I**), respectively. **J** Representative western blot and quantification of ENTPD5 expression in the kidneys of *db/db* mice at 16, 24, 32, and 40 weeks (*n* = 4 per group). **K**, **M** Representative western blot (WT, *n* = 3 and *db/db*, *n* = 4 blots) and quantification of ENTPD5 expression in the kidneys of *db/db* mice at 16 (**K**) and 40 weeks (**M**). **L**, **N** Relative mRNA levels of ENTPD5 in the kidneys of *db/db* mice at 16 weeks (**L**) and 40 weeks (**N**) (*n* = 5 per group). **O**, **P** Representative IHC images and quantification of ENTPD5 in renal tissue of *db/db* mice at 16 weeks (**O**) and 40 weeks (**P**) (*n* = 5 per group). Scale bar, blue 100 μm. **Q** Functional enrichment analysis of ENTPD5 expression using KEGG pathway based on LC-MS/MS data in the kidneys of 16-week-old *db/db* mice. **R**, **S** Representative western blot and quantification of ENTPD5 expression in RTECs exposed to glucose (15 and 30 mmol/L) (**R**) and palmitic acid (PA, 0.1 and 0.2 mmol/L) (**S**) (*n* = 3 blots). Data are mean ± SD. *P < 0.05, **P < 0.01, ***P < 0.001, ****P < 0.0001.

ENTPD5, a nucleotide hydrolase located in the ER, hydrolyzes UDP to UMP. In this study, we found that ENTPD5 was expressed in kidney and liver tissues but not in the spleen or myocardial tissues in humans (Fig. 1A), and according to the Genecards database (http://www.genecards.org), ENTPD5 is highly expressed in proximal renal tubules. Considering the specific expression of ENTPD5 in the kidney, we examined the role of ENTPD5 in DKD. To determine whether ENTPD5 is associated with DKD pathophysiological progression, immunohistochemical (IHC) staining showed that ENTPD5 was mainly expressed in proximal renal tubules in renal biopsy samples taken from DKD patients. Surprisingly, this expression showed dynamic alterations; that is, pathological diagnosis revealed that ENTPD5 levels were increased in the biopsy samples from patients with class I DKD but decreased in class II, III, and IV samples (Fig. 1B). Dynamic alterations in transcription were observed via FISH (Fig. 1C). We studied other proteinuric nephropathies and found that the protein and transcriptional expression of ENTPD5 was increased in minimal glomerular lesions (minimal change disease, MCD) but was decreased in sclerosing glomerulonephritis (SGN) (Fig. 1D, E). Notably, no correlation was found between ENTPD5 and urinary albumin or lipid levels (Fig. 1F, G); however, in all patients, ENTPD5 levels were negatively correlated with serum creatinine and were positively correlated with eGFR (Fig. 1H, I), which are key indicators of kidney damage. These observations suggest that ENTPD5 may be related to renal disease.

Similarly, ENTPD5 protein levels were significantly higher in the kidneys of *db/db* diabetic mice at 16 weeks, 24 weeks, and 32 weeks and were significantly lower at 40 weeks than in the wild-type group (Fig. 1J). The mRNA and protein levels of ENTPD5 were higher in the kidney tissues of 16-week-old *db/db* mice (Fig. 1K, L) and lower in 40-week-old *db/db* mice (Fig. 1M, N); similar results were obtained by IHC staining of the kidney tissues from *db/db* mice (Fig. 1O, P). Liquid chromatography with tandem mass spectrometry (LC‒MS/MS) protein profiling of kidney tissue from 16-week-old *db/db* diabetic mice revealed that ENTPD5 was enriched in multiple pathways (Fig. 1Q), indicating that ENTPD5 may play an important role in regulating metabolic events.

Since ENTPD5 was mainly expressed in proximal renal tubules, the expression of ENTPD5 was further examined in renal tubule epithelial cells (RTECs) under pathophysiological conditions. In vitro, ENTPD5 protein levels were significantly increased in RTECs exposed to low concentrations of glucose or palmitic acid (PA) (15 mM glucose and 0.1 mM PA), while ENTPD5 expression was decreased in RTECs exposed to high concentrations of glucose (HG) or PA (30 mM glucose and 0.2 mM PA) compared with those in the control group after long-term culture (Fig. 1R, S). These results were consistent with those showing dynamic ENTPD5 expression in DKD; ENTPD5 levels were increased in the early stage and then decreased with persistent hyperglycemia and hyperlipidemia in the late stage, suggesting that high ENTPD5 expression may alleviate kidney injury and that the downregulation of ENTPD5 expression is closely associated with the pathophysiological progression of DKD.

### Decreased ENTPD5 in RTECs exacerbates renal injury in diabetic mice

**Fig. 2:**
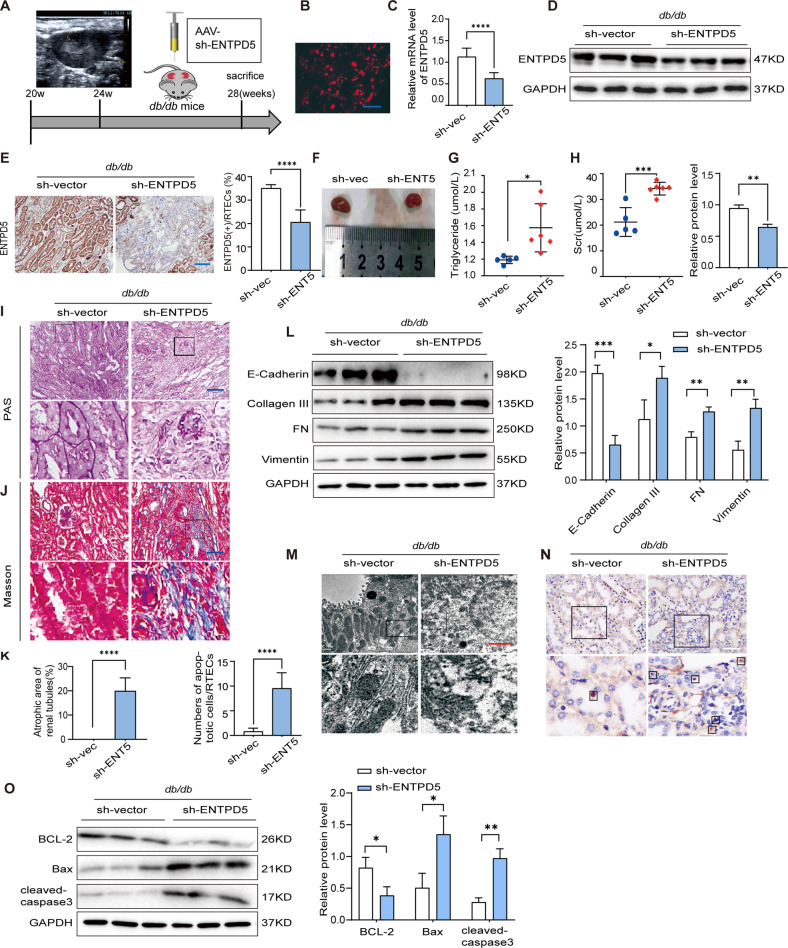
Decreased ENTPD5 in RTECs exacerbates renal injury in diabetic mice. **A** Beginning at 20 weeks of age, male *db/db* mice were followed multipoint injection in situ of ENTPD5 knockdown adeno-associated virus (AAV-Sh-ENTPD5) or AAV-vector by using ultrasound and continue feeding to 28 weeks to sacrifice. **B** Representative fluorescent images of adeno-associated in the kidney of *db/db* mice after injection AAV-Sh-ENTPD5. Scale bar, blue 100 μm. **C**, **D** Relative mRNA expression levels (*n* = 3 per group) and representative western blot and quantification (*n* = 3 blots) of ENTPD5 in the kidney of *db/db* mice with AAV-Sh-ENTPD5 virus. **E** Representative IHC images and quantification of ENTPD5 in renal tissue of *db/db* mice with AAV-Sh-ENTPD5 virus (*n* = 5 per group). Scale bar, blue 100 μm. **F** Kidney morphology of *db/db* mice injected with AAV-Sh-ENTPD5 virus at 28 weeks (*n* = 5 per group). **G**, **H** Serum triglycerides (**G**) and creatinine (**H**) in *db/db* mice (WT, *n* = 5; *db/db*, *n* = 6) injected with AAV-Sh-ENTPD5 virus at 28 weeks. **I**, **J** Representative PAS (**I**) and Masson staining (**J**) images in the kidneys of *db/db* mice injected with AAV-Sh-ENTPD5 virus (*n* = 3 per group). Scale bar, blue 100 μm. **K** Atrophic area of renal tubules in the kidneys of *db/db* mice injected with AAV-Sh-ENTPD5 virus, calculated by PAS staining. **L** Representative western blot and quantification of EMT- and ECM-related proteins (E-cadherin, Collagen III, FN, and Vimentin) expression in the kidneys of ENTPD5 knockdown *db/db* mice (*n* = 3 blots). **M** Representative transmission electron microscopy images of kidney from ENTPD5 knockdown *db/db* mice (*n* = 6 images). Scale bar, red 100 nm. **N** Representative TUNEL staining images and quantification of apoptosis in the kidney of ENTPD5 knockdown *db/db* mice (*n* = 3 per group). Scale bar, white 50 μm. **O** Representative western blot and quantification of apoptosis-related protein (BCL-2, Bax and Caspase-3) in the kidneys of ENTPD5 knockdown *db/db* mice (*n* = 3 blots). Data are mean ± SD. *P < 0.05, **P < 0.01, ***P < 0.001, ****P < 0.0001.

Considering the dynamic expression of ENTPD5 in the DKD kidney, we altered ENTPD5 expression in the early stage of DKD, since ENTPD5 was upregulated in this stage. We generated an ENTPD5-specific knockdown mouse model through multipoint injection of adeno-associated virus expressing short hairpin ENTPD5 (AAV-sh-ENTPD5) into the renal cortex in *db/db* mice subjected to B-mode ultrasound (Fig. 2A). Intense red fluorescence was observed in the renal cortex of the kidney (Fig. 2B), and the mRNA and protein levels of ENTPD5 were significantly reduced compared with those in the control group, as determined by qPCR, western blot analysis and IHC staining (Fig. 2C–E), indicating that AAV-sh-ENTPD5 was specifically delivered to the renal cortex and sufficiently inhibited ENTPD5 expression. The kidneys were smaller in ENTPD5-knockdown mice than in control mice (Fig. 2F). The serum levels of triglycerides and creatinine were increased in ENTPD5-knockdown *db/db* mice (Fig. 2G, H). Masson’s staining and periodic acid Schiff (PAS) staining revealed exacerbated renal tubular damage, as indicated by multifocal atrophy in renal tubules, multifocal fibrosis in the renal interstitium, and chronic infiltration of inflammatory cells in ENTPD5-knockdown *db/db* mice (Fig. 2I, J). The minimum shrinkage area of the renal tissue was ~13.59%, and the maximum area was ~25.52% compared with those in the control group (Fig. 2K), as calculated by PAS staining.

Furthermore, the levels of epithelial-mesenchymal transition (EMT)- and extracellular matrix (ECM)-related proteins in the kidney tissues of ENTPD5-knockdown *db/db* mice were increased (Fig. 2L). Notably, the number of mitochondria and endoplasmic reticula in RTECs was significantly reduced in the kidneys of ENTPD5-knockdown *db/db* mice, as indicated by transmission electron microscopy (TEM) (Fig. 2M). Importantly, TUNEL staining indicated that the apoptosis rates of RTECs were dramatically increased (Fig. 2N), and similar results were determined by Western blot analysis of proteins extracted from the kidneys of ENTPD5-knockdown *db/db* mice; specifically, the levels of proapoptotic proteins (Bax, cleaved-caspase-3) were increased, and the levels of antiapoptostic proteins (BCL-2) were decreased (Fig. 2O).

These results suggested that ENTPD5 downregulation in the early stage of DKD exacerbated renal tubular damage and induced cell apoptosis, thus accelerating tubular atrophy and renal interstitial fibrosis in the kidney.

### ENTPD5 drives proliferation or apoptosis in RTECs under diabetic conditions

**Fig. 3:**
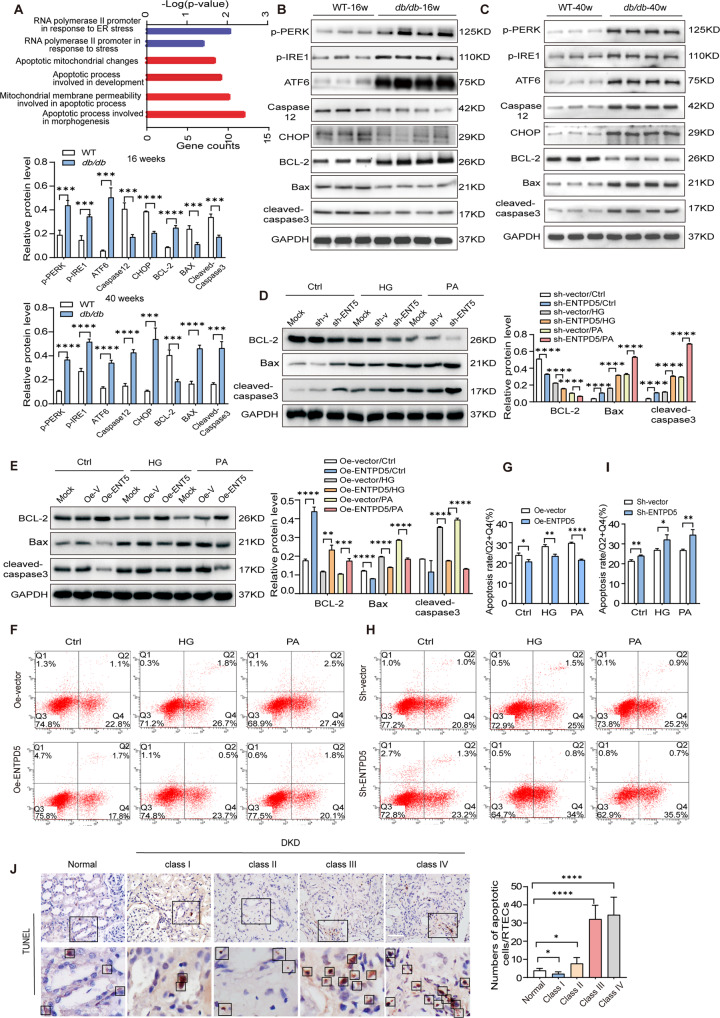
ENTPD5 drives proliferation or apoptosis in RTECs under diabetic conditions. **A** Pathway enrichment analysis in control or ENTPD5 overexpressing RTECs, based on RNA-Seq data (*n* = 3 per group). **B**, **C** Representative western blot and quantification of ER stress-related proteins (*p*-PERK, *p*-IRE1, ATF6) and apoptosis-related protein (Caspase12, CHOP, BCL-2, Bax, and Caspase-3) in the kidneys of 16-week-old *db/db* mice (**B**) and 40-week-old *db/db* mice (**C**) (*n* = 3 blots). **D**, **E** Representative western blot and quantification of apoptosis-related protein expression (BCL-2, Bax, and Caspase-3) in ENTPD5 knockdown (**D**) and overexpressing (**E**) RTECs exposed to HG (30 mmol/L) and PA (0.2 mmol/L) for 48 h, respectively (*n* = 3 blots). **F**, **G** Annexin V-PE/7-AAD double staining images (**F**) and apoptosis rate (**G**) in ENTPD5 overexpressing RTECs exposed to HG (30 mmol/L) or PA (0.2 mmol/L) for 48 h, using flow cytometry assay (*n* = 3 per group). **H**, **I** Annexin V-PE/7-AAD double staining images (**H**) and quantification (**I**) of apoptosis rate in ENTPD5 knockdown RTECs exposed to HG (30 mmol/L) and PA (0.2 mmol/L) for 48 h, respectively (*n* = 3 per group). **J** Representative TUNEL images of kidney tissues from DKD patients and quantification of renal tubular apoptosis rate. Scale bar, white 50 μm. Data are mean ± SD. *P < 0.05, **P < 0.01, ***P < 0.001, ****P < 0.0001.

Based on the observation that ENTPD5 was closely related to DKD progression, functional investigations were carried out to examine the manner in which ENTPD5 is involved in renal injury in DKD. Gene expression in RTECs was measured by RNA sequencing (RNA-Seq) and the results revealed 750 upregulated genes and 920 downregulated genes in ENTPD5-overexpressing cells. These genes are primarily involved in ER stress and apoptosis signaling pathways (Fig. 3A). Therefore, we hypothesized that ENTPD5 may play an important role in regulating ER stress and apoptosis.

The compensatory proliferation of renal tubular cells has been reported to contribute to hypertrophy, and the loss of compensatory proliferation has been suggested to result in apoptotic atrophy in DKD [4, 5]. We found that the cell proliferation rate was increased and that the number of apoptotic cells was decreased, as determined by TUNEL and hematoxylin and eosin (HE) and Masson staining, in the early stage of 16-week-old *db/db* mice (Supplementary Fig. 1A, B). We examined pathological changes in glomeruli, and increased glomerular volume, narrowed renal capsule lumen, irregular thickening of the glomerular basement membrane, and fusion of the foot process basement membrane were observed in 16-week-old *db/db* mice, as indicated by PAS staining and TEM (Supplementary Fig. 1C, D). In addition, ER stress was activated in renal tubular cells (Fig. 3B), as characterized by decreased protein levels of the apoptosis-regulated protein C/EBP homologous protein (CHOP) (Fig. 3B). While ER stress and apoptosis pathways were activated in the kidneys of 40-week-old *db/db* mice in end-stage DKD (Fig. 3C), and the cell proliferation rate decreased while the number of apoptotic cells increased (Supplementary Fig. 1E); in addition, the kidney exhibited tubular atrophy and interstitial fibrous tissue hyperplasia (Supplementary Fig. 1F). However, PAS staining and TEM analysis showed heavy hyperplasia in focal segments, marked widening of the thylakoid zone and thickening of the basement membrane, and marked fusion of podocyte peduncles in *db/db* mice at 40 weeks compared to WT mice (Supplementary Fig. 1G, H). TEM showed that the numbers of mitochondria and endoplasmic reticula in RTECs were significantly reduced in the kidneys of 40-week-old *db/db* mice (Supplementary Fig. 1I), while the numbers of mitochondria and endoplasmic reticula were increased in the kidneys of 16-week-old *db/db* mice (Supplementary Fig. 1J). In vitro, low concentrations of glucose (15 mmol/L) and PA (0.1 mmol/L) promoted proliferation and inhibited apoptosis in RTECs, while HG (30 mmol/L) and PA (0.2 mmol/L) led to the opposite effects (Supplementary Fig. 1K–M), suggesting that compensatory proliferation and decompensatory apoptosis in RTECs contributed to DKD progression.

Combining the dynamic expression pattern of ENTPD5 in DKD with the RNA-Seq data, we sought to determine whether ENTPD5 was involved in the process that begins with hypertrophy and progresses to RTEC apoptosis in DKD. We generated stable cell lines with ENTPD5 overexpression and knockdown via lentivirus. The efficiency of lentiviral-induced ENTPD5 overexpression and knockdown were significant (Supplementary Fig. 1N). Western blot analysis showed that the apoptosis pathway was significantly activated (as indicated by the increased Bax and cleaved caspase-3 levels and reduced BCL-2 levels) in ENTPD5-knockdown RTECs exposed to HG or high concentrations of PA (Fig. 3D). In contrast, ENTPD5 overexpression inhibited apoptosis pathway activation (Fig. 3E). Cell apoptosis was further analyzed by flow cytometry, and the results showed that fewer RTECs overexpressing ENTPD5 underwent apoptosis, and a higher number of ENTPD5-knockdown cells underwent apoptosis (Fig. 3F–I), suggesting that ENTPD5 participates in apoptosis in DKD. Furthermore, we found that the apoptosis rate of RTECs was decreased in class I DKD patients, while the apoptosis rate was increased in class II, III, and IV DKD patients, which negatively correlated with ENTPD5 expression in the kidneys of DKD patients (Fig. 3J).

Overall, ENTPD5 may regulate RTEC proliferation in a compensatory manner to adapt to the metabolic environment in the early stage of DKD. As the duration and size of the lesions increase, however, a reduction in ENTPD5 levels leads to increased RTEC apoptosis, causing renal tubular injury, tubular atrophy, and interstitial fibrosis in the kidney.

### ENTPD5 regulates ER stress-mediated cell proliferation and apoptosis through protein N-glycosylation

**Fig. 4:**
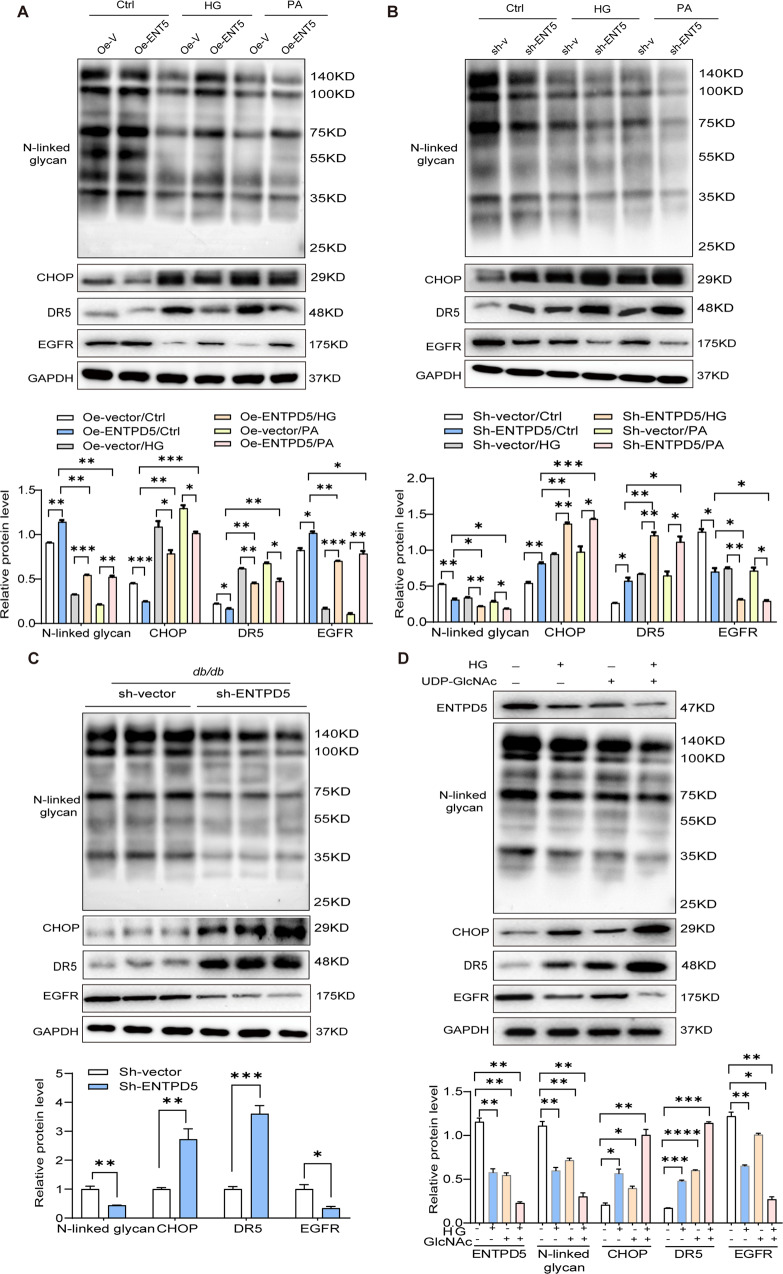
ENTPD5 regulates ER stress-mediated cell proliferation and apoptosis through protein N-glycosylation. **A** Representative western blot and quantification of protein expression (N-GlcNAc, CHOP, DR5, and EGFR) in ENTPD5 overexpressing RTECs exposed to HG (30 mmol/L) and PA (0.2 mmol/L) for 48 h (*n* = 3 blots). **B** Representative western blot and quantification of protein expression (N-GlcNAc, CHOP, DR5, and EGFR) in ENTPD5 knockdown RTECs exposed to HG (30 mmol/L) and PA (0.2 mmol/L) for 48 h (*n* = 3 blots). **C** Representative western blot and quantification of protein expression (N-GlcNAc, CHOP, DR5, and EGFR) in the kidneys of ENTPD5 knockdown *db/db* mice (*n* = 3 blots). **D** Representative western blot and quantification of protein expression (ENTPD5, N-GlcNAc, DR5, CHOP, and EGFR) in RTECs exposed to NG (5.5 mmol/L) and HG (30 mmol/L) plus N-glycosylated substrate UDP-GlcNAc (10 mmol/L) for 48 h (*n* = 3 blots). Data are mean ± SD. *P < 0.05, **P < 0.01, ***P < 0.001, ****P < 0.0001.

Further analysis of the RNA-Seq data of RTECs overexpressing ENTPD5 revealed that CHOP, an ER stress-specific transcription factor, transcriptionally regulates death receptor 5 (DR5) expression, thereby activating exogenous apoptotic pathways to mediate apoptosis [18, 19]. Western blot analysis showed that the protein levels of CHOP and DR5 were reduced in ENTPD5-overexpressing RTECs, generally in response to HG- or PA-induced CHOP and DR5 expression, indicating that ENTPD5 was negatively correlated with CHOP and DR5 expression (Fig. 4A), while CHOP and DR5 levels were elevated in ENTPD5-knockdown RTECs in the presence or absence of HG or PA (Fig. 4B).

Proteins modified by N-GlcNAc play key roles in the maturation and quality control of proteins, and N-glycosylation deficiency induces UPR and cell apoptosis [6]. In addition, N-GlcNAc is critical for growth factor receptor translocation to the plasma membrane [7]. To clarify the specific mechanism by which ENTPD5 regulates ER stress, we performed a lectin-binding assay using concanavalin A (Con A), which specifically detects proteins with the N-GlcNAc modification by binding to Asn-linked glycans in the candidate protein. HG and PA treatment reduced the levels of proteins modified with N-GlcNAc in RTECs. In contrast, ENTPD5 significantly increased the level of protein glycosylation (Fig. 4A). In addition, the level of EGFR, a representative cell surface receptor that regulates cell proliferation, was significantly increased (Fig. 4A). Conversely, ENTPD5 knockdown inhibited protein glycosylation and reduced EGFR expression levels in RTECs (Fig. 4B). Importantly, the abundance of protein glycosylation and EGFR expression levels were greatly reduced in the kidneys of ENTPD5-knockdown *db/db* mice in vivo, but CHOP and DR5 expression levels were significantly increased (Fig. 4C).

Moreover, we used UDP-GlcNAc, an essential substrate of N-GlcNAc, to treat RTECs cultured with normal glucose (NG, 5.5 mmol/L) and HG (30 mmol/L) and found that the expression levels of ENTPD5, N-GlcNAc, and EGFR were decreased, while the expression of CHOP and DR5 was increased. When RTECs were treated with UDP-GlcNAc plus HG, these outcomes were amplified (Fig. 4D). These results indicated that high concentrations of UDP-GlcNAc, particularly in a HG environment, inhibited protein glycosylation and activated ER stress.

Because protein N-GlcNAc is tightly regulated by the amount of UDP-glucose transported to the ER, UMP/CMP kinase1 requires UMP to produce the UDP required for UDP-glucose generation. However, UMP production is mediated by ENTPD5 primarily through UDP hydrolyzation. As mentioned previously, even in the presence of a high concentration of UDP-GlcNAc, the level of proteins with the N-GlcNAc modification was inhibited (Fig. 4D), which was probably due to a decrease in UDP-glucose transported to the ER. Specifically, UDP-glucose transport to the ER is facilitated by an antiporter in conjunction with UMP export from the ER lumen; however, decreased ENTPD5 levels result in insufficient UMP, which inhibits protein glycosylation. Taken together, these data suggested that ENTPD5 strictly regulates the levels of proteins with the N-GlcNAc modification, triggering CHOP-induced apoptosis and EGFR-induced proliferation of RTECs in the kidney.

### SP1 regulates the expression of ENTPD5 under diabetic conditions

**Fig. 5:**
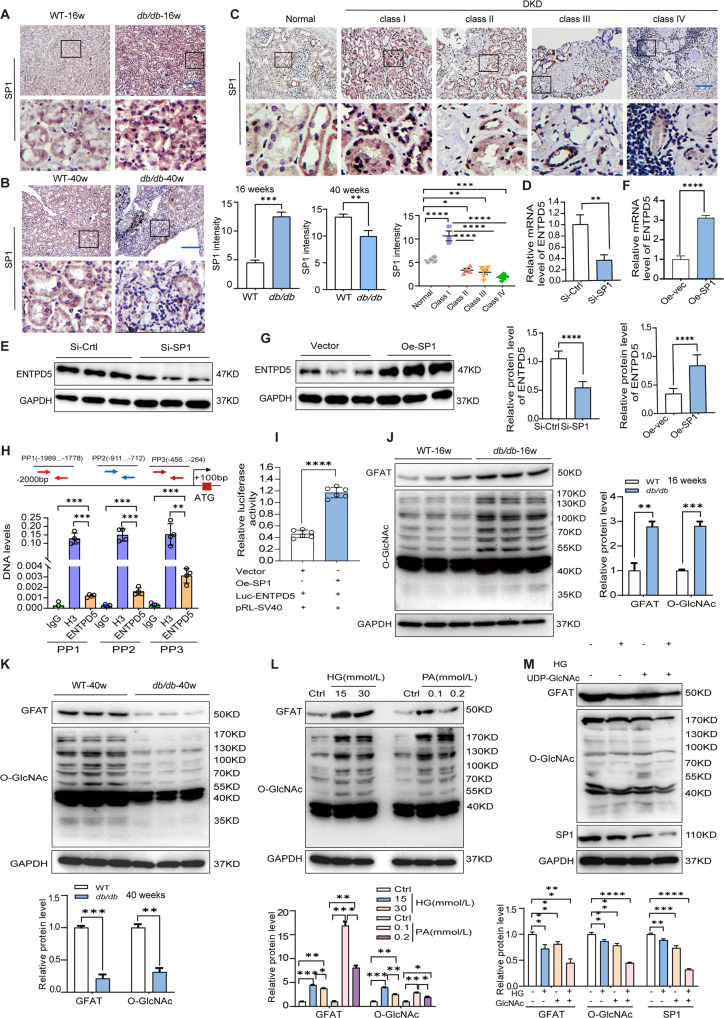
SP1 regulates the expression of ENTPD5 under diabetic conditions. **A**, **B** Representative IHC images and quantification of SP1 expression in the kidneys of 16-week-old *db/db* mice (**A**) and 40-week-old *db/db* mice (**B**) (*n* = 5 per group). Scale bar, blue 100 μm. **C** Representative IHC images and quantification of proximal tubular SP1 expression in the kidney of DKD patients (grading and the number of cases as in Fig. 1B). Scale bar, blue 100 μm. **D**, **E** Relative mRNA levels (**D**) and representative western blot (*n* = 3 blots) and quantification of ENTPD5 expression (**E**) in SP1 knockdown RTECs. **F**, **G** Relative mRNA levels (**F**) and representative western blot (*n* = 3 blots) and quantification of ENTPD5 expression (**G**) in SP1 overexpressing RTECs. **H**, **I** ChIP assay (**H**) and dual-luciferase assay (**I**) to verify the regulation of transcript levels of ENTPD5 by SP1 (*n* = 3 per group); normal rabbit IgG as a negative control antibody and rabbit Histone H3 as a positive antibody. **J**, **K** Representative western blot and quantification of protein expression (GFAT, O-GlcNAc) in the kidneys of 16-week-old *db/db* mice (**J**) and 40-week-old *db/db* mice (**K**) (*n* = 3 blots). **L** Representative western blot and quantification of protein expression (GFAT, O-GlcNAc expression in RTECs exposed to HG (15 and 30 mmol/L) and PA (0.1 and 0.2 mmol/L) (*n* = 3 blots). **M** Representative western blot and quantification of protein expression (GFAT, O-GlcNAc and SP1) in RTECs exposed to HG (30 mmol/L) plus UDP-GlcNAc (10 mmol/L) for 48 h. (*n* = 3 blots). Data are mean ± SD. *P < 0.05, **P < 0.01, ***P < 0.001, ****P < 0.0001.

To clarify the mechanism by which ENTPD5 is dynamically expressed in RTECs in DKD, we focused on the transcription factor SP1, which regulates the transcription of ENTPD5. IHC staining showed that SP1 was distributed in the cytoplasm and nucleus in RTECs, and interestingly, increased SP1 expression in the cytoplasm and nucleus was observed in the kidneys of 16-week-old *db/db* mice (Fig. 5A). In contrast, decreased SP1 expression was observed in the kidneys of 40-week-old *db/db* mice (Fig. 5B). More importantly, an increase in the SP1 level was observed in the cytoplasm and nucleus of renal tubules in class I DKD patients (Fig. 5C). However, with the progression of the lesion, the expression of SP1 in the cytoplasm and nucleus gradually decreased in class II, III and IV DKD patients (Fig. 5C). In addition, the expression of SP1 was increased in RTECs cultured with lower concentrations of glucose or PA but was decreased in RTECs cultured with HG or high concentrations of PA (Supplementary Fig. 2A). Thus, the dynamic expression of SP1 in the kidney tissue of DKD paralleled the expression pattern of ENTPD5; that is, the levels of both proteins first increased and then gradually decreased. Moreover, SP1 knockdown decreased the mRNA and protein levels of ENTPD5 (Fig. 5D, E) while SP1 overexpression increased ENTPD5 mRNA and protein levels (Fig. 5F, G). Chromatin immunoprecipitation (ChIP)-qPCR analysis clearly demonstrated that SP1 bound to the promoter region of the ENTPD5 gene (Fig. 5H) and this binding was confirmed with a dual-luciferase reporter assay system (Fig. 5I), suggesting that SP1 directly regulates the transcription of ENTPD5.

Next, we examined how SP1 dynamically regulates the expression of ENTPD5. SP1 activity is mediated via O-glycosylation (with O-GlcNAc), which determines the nuclear translocation and stability of SP1 [20, 21]. Low levels of O-glycosylated SP1 are preferentially degraded via the proteasome to inhibit transcriptional activity. Hyperglycemia has been shown to increase the degree of SP1 modification with O-GlcNAc through the hexosamine biosynthesis pathway (HBP) [22–24].

Consistent with this finding, the levels of O-GlcNAc and glutamine-fructose-6-phosphate aminotransferase (GFAT), which is the rate-limiting enzyme in the HBP [25, 26], were increased in the kidneys of 16-week-old *db/db* mice (Fig. 5J) and decreased in *db/db* mice at 40 weeks (Fig. 5K). Similarly, O-GlcNAc abundance and GFAT expression were increased in RTECs cultured with low concentrations of glucose or PA, while high concentrations of HG or PA led to the opposite outcomes (Fig. 5L). However, UDP-GlcNAc, an end-product of HBP, negatively regulates GFAT expression [25]. Our experiments revealed that a low concentration of UDP-GlcNAc exerted no inhibitory effect on GFAT expression, while a high concentration of UDP-GlcNAc significantly inhibited GFAT expression (Supplementary Fig. 2B). Importantly, the level of O-GlcNAc and the expression levels of GFAT and SP1 were decreased in RTECs cultured with HG or NG plus high concentrations of UDP-GlcNAc, and this effect was more obvious in RTECs exposed to UDP-GlcNAc plus HG (Fig. 5M).

Taken together, these results suggest that ENTPD5 expression is regulated by O-GlcNAc modification of SP1. Hyperglycemia promotes glucose use in the HBP, thus increasing the level of the end-product UDP-GlcNAc via the rate-limiting enzyme GFAT and increasing the rate of SP1 glycosylation with O-GlcNAc to promote SP1-induced transcription of ENTPD5. In hyperglycemia and DKD conditions, the continual increase in UDP-GlcNAc inhibits GFAT expression via a negative feedback mechanism, resulting in a decrease in UDP-GlcNAc levels, inhibiting SP1 modification with O-GlcNAc and, therefore, downregulating ENTPD5 expression.

### ENTPD5 negatively regulates renal injury in UUO mice

**Fig. 6:**
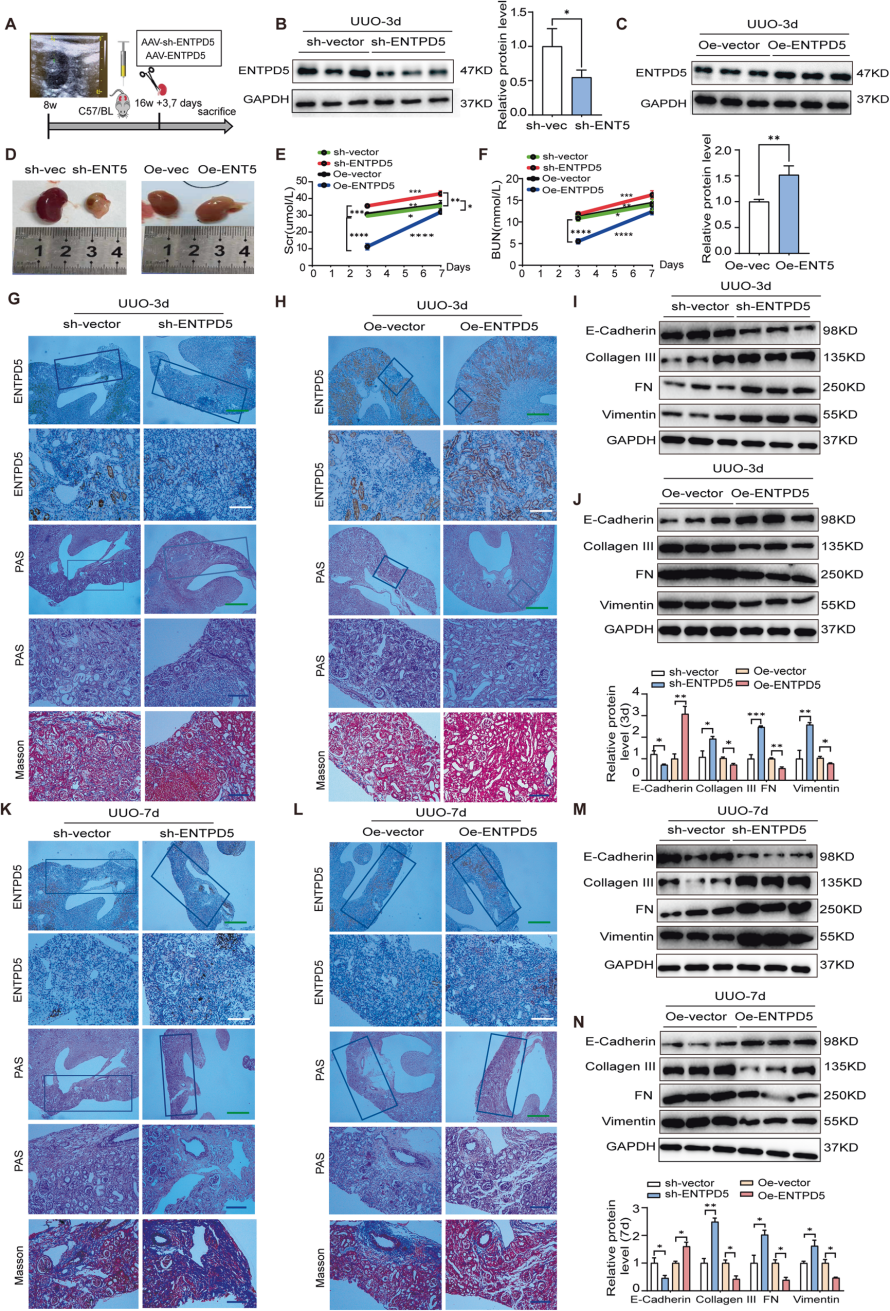
ENTPD5 negatively regulates renal injury in UUO mice. **A** Beginning at 8 weeks of age, the renal cortex of male C57BL mice was followed multipoint injection in situ of ENTPD5 knockdown or ENTPD5 overexpressing adeno-associated virus (AAV-Sh-ENTPD5, AAV-ENTPD5) or AAV-vector by using ultrasound. To operate UUO surgery at 16 weeks after the injection and the mice were executed at 3 and 7 days postoperatively. **B**, **C** Representative western blot and quantification of ENTPD5 expression in the kidneys of UUO mice with ENTPD5 knockdown (**B**) or overexpression (**C**) at 3 days postoperatively (*n* = 3 blots). **D** Kidney morphology of UUO mice at 3 days postoperatively after injection of AAV-Sh-ENTPD5 and AAV-ENTPD5 virus. **E**, **F** The level of serum creatinine (**E**) and blood urea nitrogen (BUN) (**F**) from UUO mice with ENTPD5 knockdown or overexpression at 3 and 7 days postoperatively (vector, *n* = 3 and AAV-ENTPD5 or AAV-sh-ENTPD5, *n* = 4, respectively). **G**, **H** Representative IHC images of ENTPD5 expression, PAS and Masson staining of kidney tissue from UUO mice with ENTPD5 knockdown (**G**) or overexpression (**H**) at 3 days postoperatively (vector, *n* = 3 and AAV-ENTPD5 or AAV-sh-ENTPD5, *n* = 4, respectively). Scale bar: green 500 μm, blue 100 μm and white 50 μm. **I**, **J** Representative western blot and quantification of EMT- and ECM-related proteins (E-cadherin, Collagen III, FN and Vimentin) expression in the kidney of UUO mice with ENTPD5 knockdown (**I**) or overexpression (**J**) at 3 days postoperatively (*n* = 3 blots). **K**, **L** Representative IHC images of ENTPD5 expression, PAS and Masson staining of kidney tissue from UUO mice with ENTPD5 knockdown (**K**) or overexpression (**L**) at 7 days postoperatively (vector, *n* = 3 and AAV-ENTPD5 or AAV-sh-ENTPD5, *n* = 4, respectively). Scale bar: green 500 μm, blue 100 μm, and white 50 μm. **M**, **N** Representative western blot and quantification of EMT- and ECM-related proteins (E-cadherin, Collagen III, FN, and Vimentin) expression in the kidney of UUO mice with ENTPD5 knockdown (**M**) or overexpression (**N**) at 3 days postoperatively (*n* = 3 blots).

To examine whether ENTPD5 is involved in renal injury in other kidney diseases, we used a murine model of unilateral ureteral obstruction (UUO)-induced nephropathy, which recapitulates human SGN with typical pathological changes in renal interstitial fibrosis in the end stage of chronic kidney disease. UUO mice were administered AAV-ENTPD5 and AAV-sh-ENTPD5 separately via renal cortex multipoint injection to upregulate and downregulate ENTPD5 expression, respectively (Fig. 6A). The results showed that the protein levels of ENTPD5 were significantly decreased in the kidneys of UUO mice compared with those in the control group after ENTPD5 knockdown 3 days or 7 days after UUO surgery (Fig. 6B and Supplementary Fig. 3A) and were significantly increased in UUO mice with ENTPD5 overexpression (Fig. 6C and Supplementary Fig. 3B).

Notably, kidney size in mice with ENTPD5 knockdown was smaller than that in the control group but was increased in ENTPD5-overexpressing mice 3 days after the UUO operation (Fig. 6D), and similar results were observed in mice 7 days after the operation (data not shown). Serum creatinine and blood urea nitrogen levels were significantly increased in ENTPD5-knockdown UUO mice but were significantly reduced in ENTPD5-overexpressing UUO mice on the 3rd and 7th days after the UUO operation (Fig. 6E, F). PAS and Masson’s staining revealed great improvements in renal morphology and reduced renal interstitial fibrosis after ENTPD5 was overexpressed (Fig. 6H), while pathological renal morphology and increased renal interstitial fibrosis were observed in the kidneys of ENTPD5-knockdown UUO mice 3 days after the UUO operation (Fig. 6G). Similar results were observed in mice 7 days after the operation (Fig. 6K, L). In addition, the levels of EMT- and ECM-related proteins in the kidney tissues of the UUO mice expressing AAV-ENTPD5 were reduced (Fig. 6J, N), while these protein levels were increased in UUO mice with ENTPD5 knockdown on the 3rd and 7th days after the UUO operation (Fig. 6I, M). These results suggested that ENTPD5 plays an important role in renal fibrosis in chronic kidney disease.

In addition, the TUNEL assay showed an increase in the number of apoptotic renal tubular cells in UUO mouse kidneys after ENTPD5 knockdown on the 3rd and 7th days after surgery compared to that in the control group at the same time points (Supplementary Fig. 3C, D). The apoptosis rates of renal tubular cells on the 3rd and 7th days after surgery were attenuated in UUO mice overexpressing ENTPD5 (Supplementary Fig. 3E, F). Western blot analysis demonstrated that proapoptotic protein levels were significantly decreased and antiapoptotic protein levels were increased in the kidneys of UUO mice after ENTPD5 was overexpressed (Supplementary Fig. 3G, I), and the opposite outcomes were observed in UUO mice with ENTPD5 knockdown on the 3rd and 7th days after surgery (Supplementary Fig. 3H, J). These results suggest that ENTPD5 alleviated renal injury in UUO mice and may be a potential therapeutic target to protect RTECs against injury.

## Discussion

Renal tubules exhibited hypertrophy in the early stage of DKD in mice, and the numbers of proximal and distal tubules were increased in the kidneys of mice treated with streptozotocin (STZ) to induce diabetes at 13 weeks [5], as well as in 12-week-old *db/db* mice [27, 28]. In our study, we also observed that the numbers of proximal and distal tubules were increased in the kidneys of 16-week-old *db/db* mice, indicating that renal tubule hypertrophy and hyperplasia contributed to the increased kidney size in DKD, which is considered an early pathological change in DKD. With continuous hyperglycemia, renal tubule lengths were initially increased with tubule lumen enlargement, and eventually, renal tubule cells underwent apoptosis [29, 30], which ultimately led to tubular atrophy and interstitial fibrosis; however, the observed phenomena might arise from secondary effects of the Lepr mutation in *db/db* mice. However, the mechanisms by which renal tubule pathology progresses from tissue hypertrophy to cell apoptosis and then to atrophy in diabetes mellitus are still unclear.

ENTPD5 is the only identified intracellular ENTPDase [31]. In this study, we confirmed the role of ENTPD5 in mediating ER stress to regulate adaptation and induce damage to renal tubules in DKD. Specifically, ENTPD5 was differentially expressed in the kidneys of diabetic mice, as determined by LC‒MS analysis. Functional experiments demonstrated that ENTPD5 was mainly expressed in the renal tubules of the kidney and that the levels of ENTPD5 were altered under pathological conditions, initially increasing and then decreasing in the late stage of DKD in diabetic mice and patients. Therefore, ENTPD5 can be used as a diagnostic marker to determine the pathological stage of DKD in the clinic. Importantly, ENTPD5 downregulation in RTECs significantly exacerbated kidney injury, inhibiting RTEC proliferation and promoting apoptosis in diabetic mice and UUO mice, while ENTPD5 overexpression attenuated kidney injury, suggesting that ENTPD5 is pivotal in preventing renal tubule pathology from progressing from tissue hypertrophy and cell apoptosis to atrophy and that it plays an important role in protecting renal tubules from injury.

Through mechanistic investigations, we found that ENTPD5 upregulation promoted RTEC proliferation and inhibited RTEC apoptosis, while ENTPD5 downregulation led to opposite results. In addition, we found that ENTPD5 expression was transcriptionally regulated by the transcription factor SP1 after SP1 O-glycosylation, which prevented SP1 from undergoing proteasomal degradation [24]. Glucose is metabolized through the HBP, and only 1–3% of intracellular glucose is converted to glucosamine 6-phosphate via the HBP. Notably, glucose into the HBP has been previously shown to be significantly increased with a continuous supply of intracellular glucose [32] or when glucose/lipid metabolism is dysregulated. Hyperactivity of the HBP, which is regulated by the GFAT rate-limiting enzyme, leads to an increase in the final product UDP-GlcNAc, which is the substrate for protein glycosylation with N-GlcNAc and O-GlcNAc. We confirmed that excessive UDP-GlcNAc in RTECs exerts an inhibitory effect on GFAT, which decreases the level of O-GlcNAc-modified SP1, as mediated via a feedback mechanism, and subsequently reduces ENTPD5 transcription. As previously reported, reduced ENTPD5 expression leads to reduced UDP hydrolyzation into UMP [26, 33].

ENTPD5 hydrolyzes UDP to UMP by UGGT, which relieves the inhibitory effect of UDP on protein modification with N-GlcNAc in the ER and enables greater antiporter-mediated influx of the glucose carrier UDP-glucose, which is necessary for protein modification with N-GlcNAc into the ER [34]. Moreover, the expression of the growth factor receptor EGFR is regulated by ENTPD5-mediated protein N-glycosylation and promotes cell proliferation [16, 35–37]. This finding suggested that the increase in ENTPD5 expression participated in RTEC proliferation and renal hypertrophy in the early stage of DKD. Moreover, reducing ENTPD5 levels reduced the pool of UMP-glucose available for antiporter influx of UDP-glucose into the ER, limiting the amount of substrate available for protein N-glycosylation, which led to the accumulation of unfolded or misfolded proteins in the ER and ultimately initiated the ER stress-associated apoptosis pathway. Thus, the decrease in ENTPD5 expression participated in RTEC apoptosis in the end stage of DKD.

**Fig. 7:**
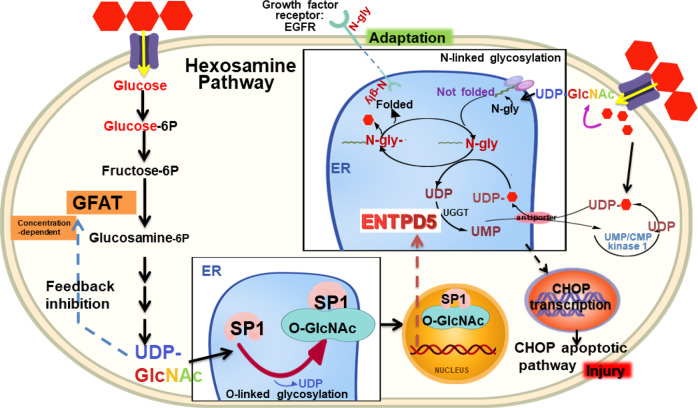
Mechanism diagram of ENTPD5 regulating protein N-glycosylation in DKD. In DKD, hyperglycemia activates the hexosamine biosynthesis pathway (HBP) to promote or inhibit SP1 O-glycosylation via a negative feedback mechanism, thus regulating ENTPD5 expression at the transcriptional level. ENTPD5 regulates unfolded protein N-glycosylation in the ER to promote cell proliferation or apoptosis of renal tubular epithelial cell.

In summary, our study first showed that ENTPD5 was important for the regulation of ER stress in RTECs from early renal hypertrophy to late apoptotic atrophy. Mechanistically, as summarized in Fig. 7, in DKD, hyperglycemia activates the HBP to promote or inhibit SP1 O-glycosylation via a negative feedback mechanism, thus regulating ENTPD5 expression at the transcriptional level. ENTPD5 regulates unfolded protein N-glycosylation in the ER to promote cell proliferation or apoptosis. Because ENTPD5 is pathophysiologically related to renal tubule injury, this work provides a new therapeutic strategy to mediate ENTPD5 expression to protect the kidney against injury in DKD or other forms of chronic kidney disease. The study also suggests that ENTPD5 may be a diagnostic marker of progressive DKD. It also explains, in part, the possible mechanism underlying the pathological changes in RTEC in DKD.

## Materials and methods

### Human renal biopsy samples

Human kidney biopsies were collected as part of routine clinical diagnostic and are shown in Supplementary Tables 1 and 2. Kidney tissue samples were obtained from the Department of Pathology, Affiliated Hospital of Guizhou Medical University. Normal control samples were obtained from healthy renal poles from patients who had undergone tumor nephrectomy without diabetes or renal disease. The investigations were conducted in accordance with the principles of the Declaration of Helsinki and were approved by the Research Ethics Committee of Guizhou Medical University (Document No.2020209). All renal biopsy specimens diagnosed with DKD were classified according to the new pathological classification provided by the Society of Renal Pathology [38].

### Animal studies

All animal experimental protocols were approved by the Ethics Committee of Guizhou Medical University (Document No.2200782) and carried out in accordance with the Guide for the Care and Use of Laboratory Animals of the National Institutes of Health. All mice (3–5 per cage) were randomly grouped and housed under standard laboratory conditions (12 h on/off; lights on at 9 am; temperature (24 °C)) with free access to water and diets.

### Establishment of *db/db* mice model

Homozygote BKS *db/db* male mice were purchased from GemPharmatech (Guangzhou, China). WT mice were used as genetic control and 10-week-old *db/db* mice were continued to be maintained on normal diets for another 6 weeks, 14 weeks, 22 weeks, and 30 weeks, respectively. At the end of the study, blood samples were collected for biochemical analysis and the kidney samples were harvested for histopathological analysis. The renal cortex was dissected for protein or RNA extraction for subsequent analysis. The physical and biochemical parameters of experimental animals are shown in Supplementary Table 3.

### UUO mice

After the 12-week-old C57BL/6 male mice (GuangDong medical laboratory animal center, China) were anesthetized and fixed, an operation of unilateral ureteral obstruction (UUO) was performed. In brief, the right side of the abdomen was opened in the middle position, and the ureter was searched along the renal hilum. The ureter was separated from the surrounding fatty tissue at the renal hilum, and the proximal and distal end of the ureter were ligated with silk threads and cut from the middle with scissors, and finally sutured.

### Generation of ENTPD5 knockdown and overexpression mice

To generate ENTPD5-specific knockdown mice model, 20-week-old male *db/db* male mice and 8-week-old C57BL/6 male mice were injected adeno-associated virus expressing short hairpin ENTPD5 (AAV-sh-ENTPD5) through renal cortex multipoint injection subjected to B-mode ultrasound with the help of ultrasound doctors. For ENTPD5-specific overexpression mice model, 8-week-old C57BL/6 male mice were injected AAV8-ENTPD5 with the above operation. 100 μL of each AAV (1 × 10^11^ pfu mL^−1^) was aspirated with a 29 G insulin syringe (Becton Dickinson, USA). After injection AAV, 20-week-old *db/db* mice were fed on normal chow diets until to 28 weeks (Supplementary Table 4). Eight-week-old C57BL were fed on normal chow diets to 16 weeks and then performed the UUO surgery.

### Cell culture and handling

Mouse proximal tubule epithelial cells RTEC were obtained from ATCC and cultured in DMEM (1.0 g/L glucose) containing 10% FBS and penicillin/streptomycin. All cells requiring intervention were synchronously quiescent after 6 h in a serum-free medium and then treated with different stimuli as follows: high glucose (HG, 15 and 30 mmol/L D-glucose in the medium, 5.5 mmol/L glucose as a control), palmitic acid (PA, 0.1 and 0.2 mmol/L) or UDP-GlcNAc (10 mmol/L). Tool cells HEK293T cells were obtained from ATCC and were cultured in DMEM (4.5 g/L glucose) containing 10% FBS.

### Histological analysis of kidney tissue

Renal biopsy and the mouse kidneys were embedded in paraffin and cross-sectioned (3 μm) for histology examination. Hematoxylin–eosin staining (HE) and immunohistochemistry (IHC) analysis and were performed according to the routine procedures. Periodic acid schiff (PAS) and Masson staining were performed according to manufacturers’ instructions by using their staining kits (Solarbio, China). Slices were photographed with Olympus BX53 microscope (Olympus, Japan) and the staining of positive areas in the renal tubules was quantified with ImageJ software.

### RNA in situ hybridization

The mRNA expression of ENTPD5 in the kidney was detected by the FISH detection kit (GenePharma, China), according to the manufacturer’s instruction. In brief, paraffin slice of kidney tissues was dewaxed, digested, denatured and hybridized. The images were acquired by a FV3000 laser scanning confocal microscopy.

### RNA isolation and real-time qRT-PCR

RNA of tissues or cells was extracted with TRIzol reagent (Invitrogen, USA), and then reverse-transcribed into cDNA using the PrimeScriptRT Master Mix (Yeasen, China) according to the manufacturer’s instructions. qRT-PCR was performed using Hieff UNICON Universal Blue qPCR SYBR Green Master Mix (Yeasen, China). Fold change in gene expression normalized to GAPDH was calculated by the ∆∆CT method using Equation 2-∆∆CT. The results were shown as fold changes compared to the control group. The primers for target genes in this study are shown in Supplementary Table 5.

### Western blot

Tissues or cultured cells were lysed with RIPA buffer containing protease inhibitors, PMSF and phosphatase inhibitors. The tissues were lysed with homogenizer. The tissue or cell lysate were incubated on ice for 40 min with shaking and centrifuged. The soluble supernatant was carefully transferred to fresh EP tubes for protein assay using the BCA protein assay. Proteins were separated by 10% or 15% SDS-PAGE and transferred to PVDF membranes. The selected proteins are detected with antibodies summarized in Supplementary Table 6.

### Detection of glycoprotein with concanavalin A (Con A-HRP)

The protein transferred to the PVDF membrane was blocked with 0.5% Tween 20-PBS for 5 min, then Con A-HRP complex with a final concentration of 5 μg/mL was added, incubated at 4 °C for 16 h, and washed with PBS twice for 5 min each time. The bands were incubated with ECL luminescent solution and exposed by Tanon chemiluminescent imaging system.

### Biochemical analysis of serum samples

Serum creatinine, urea nitrogen, and triglycerides were determined by BS-240VET veterinary biochemical automatic analyzer (Mindray, China).

### Transmission electron microscopy

Electron microscopic sample handling and detection were performed by the affiliated Hospital of Guizhou Medical University. The images were collected and analyzed under transmission electron microscope (H-7500, Japan).

### TUNEL assay

Paraffin-embedded tissue sections of the kidney were stained in situ with a detection kit (Kgi Biotechnology, China) following the manufacturer’s protocols. The apoptosis rate was made by randomly counting TUNEL-positive cells in each renal cortex.

### Flow cytometry

Cell apoptosis was determined with the kit according to the manufacturer’s instructions (BD, USA). The cells were collected and labeled by fluorescein isothiocyanate (FITC)-conjugated Annexin V and propidium iodide (PI) staining.

### Lentivirus-mediated gene interference and overexpression

Gene overexpression was achieved by pHJLV004-CMV-MCS-EF1-ZsGreen-T2A-puro vector and the silencing of genes was conducted by hU6-MCS-CMV-GFP-SV40 vector, both mediated by lentivirus expression system. For virus preparation, 293T cells were transfected with lentiviral skeleton and helper plasmids (pMD2.G and psPAX2) for 72 h, and the medium supernatant was collected and concentrated. The sequences of shRNA oligonucleotides were as follows: mouse ENTPD5 (GATGGGTCCTATGAAGGCATA).

### Knockdown of siRNA mediated

Cells were cultured in medium without antibiotics. Short interfering RNA (siRNA) or control of the target gene was transfected into cells by FAM-siRNA kit (Sangon Biotech, China) according to the manufacturer’s protocol.

### Mass spectrometry analysis

Kidney tissues of 16-week *db/db* mice and wild-type mice were collected to perform proteomic analysis. A tandem mass spectrometry (MS/MS) analysis was performed at Xiamen University (Fujian, China). Protein was extracted, digested, and labeled using TMT reagent according to the manufacturer’s instructions (Thermo Scientific). The MS raw data were searched using the MASCOT engine (Matrix Science, London, UK; version 2.2) embedded into Proteome Discoverer 1.4 software for identification and quantitation analysis.

### RNA-sequencing analysis

Lysis of RTEC cells overexpressing ENTPD5 or control were collected to perform RNA-sequencing analysis. The library construction and sequencing were performed at Beijing Novogene (Beijing, China).

### Chromatin immunoprecipitation (ChIP) assay

A ChIP assay was performed using a kit (Cell Signaling Technology, USA) according to the manufacturer’s instructions. In brief, DNA-protein complexes were cross‐linked using 1% formaldehyde for 15 min. The cross‐linked chromatin samples were isolated from the cell lysates by nuclease digestion (37 °C for 20 min) and ultrasound (20% W, over 5 s, stop for 10 s, over 30–40 times in total). SP1 was immunoprecipitated using a SP1 antibody or control antibody (rabbit IgG) and then DNA was extracted. For quantitative PCR, ChIP DNA was amplified using primers of ENTPD5 promoter (Supplementary Table 5) by qPCR SYBR Green Master Mix.

### Dual-luciferase reporter assay

In brief, the ENTPD5 promoter was cloned into pGL3-Basic vector as a luciferase reporter plasmid and Rinilla luciferase (phRL-TK) was used as a reference gene. Luciferase reporter plasmid and phRL-TK vector were transfected into 293T cells for 48 h, then cell lysis was measured by the kit (Promega, USA) according to the manufacturer’s instructions. Values represent the ratio of firefly luciferase reaction intensity to internal reference Renilla luciferase reaction intensity.

### Statistical analysis

Experimental data were presented as mean ± standard deviation (SD) with GraphPad Prism 8.0. Statistical significance between two groups was assessed using Student’s *t* tests or among multiple groups using two-way ANOVA. *P* < 0.05 was considered statistically significant.

## Supplementary information

aj-checklist

Supplementary Tables

Supplementary Fig.1

Supplementary Fig.2

Supplementary Fig.3

Supplementary Figure legends

Original Data File

## Data Availability

Data and resources are available from the corresponding authors.
