# Supplementary material for: The UDPase ENTPD5 regulates ER stress-associated renal injury by mediating protein N-glycosylation
Source: Cell Death Dis. 2023 Feb 27;14(2):166. doi: 10.1038/s41419-023-05685-4 (PMC9971188; doi:10.1038/s41419-023-05685-4)
Supplement: Supplementary file 2 — Supplementary Tables [file 41419_2023_5685_MOESM2_ESM.docx]

**Supplementary Table 1. Clinical laboratory parameters in the serum of DKD patients. Related to Figure 1.**

| **Test items** | **Normal** | **DKD** |
| --- | --- | --- |
| FBG (mmol/L) | 4.92 ± 0.43 | 7.58 ± 3.96* |
| SCr (μmol/L) | 69.67 ± 17.07 | 242.5 ± 235.6* |
| BUN (mmol/L) | 4.54 ± 1.28 | 12.2 ± 9.11* |
| eGFR（ml/min） | 102.33 ± 14.83 | 51.96 ± 38.51* |
| CHO (mmol/L) | 4.28 ± 0.54 | 4.80± 1.63 |
| TG (mmol/L) | 1.04 ± 0.10 | 2.47 ± 2.89* |
| LDL-C (mmol/L) | 2.61 ± 0.59 | 2.85 ± 0.73 |
| HDL-C (mmol/L) | 1.35 ± 0.10 | 1.18 ± 0.34 |
| HbAlc (%) | NA | 7.16 ± 1.72 |

FBG, fasting blood glucose; SCr, serum creatinine; BUN, blood urea nitrogen; eGFR, estimated glomerular filtration rate; CHO, cholesterol; TG, triglyceride; LDL-C, low-density lipoprotein cholesterol; HDL-C, high-density lipoprotein cholesterol; HbAlc, hemoglobin Alc; NA, not available.

Data are expressed as mean ± SD. **P* < 0.05 vs. normal subjects.

**Supplementary Table 2. Clinical characteristics of patients****. Related to Figure 1.**

| Control group (n = 15) for serum samples analysis | | | | | | | | | | | | |
| --- | --- | --- | --- | --- | --- | --- | --- | --- | --- | --- | --- | --- |
| Number | Age (yr) | Sex | SCr  (μmol/L) | BUN  (mmol/ L) | UPR/24h (g) | FBG  (mmol/ L) | HbAlc (%) | CHO  (mmol/ L) | TG  (mmol/L) | HDL-C  (mmol/L) | LDL-C  (mmol/L) | eGFR（ml/min） |
| 1 | 43 | F | 66 | 4.37 | NA | 5.01 | NA | 4.1 | 0.74 | 1.44 | 2.63 | 98.16 |
| 2 | 83 | M | 76 | 8.05 | NA | 4.77 | NA | 5 | 1.57 | 1.29 | 3.38 | 80.20 |
| 3 | 50 | F | 63 | 4.01 | NA | 5.29 | NA | 4.92 | 0.89 | 1.45 | 3.45 | 98.86 |
| 4 | 51 | M | 93 | 5.22 | NA | 5.39 | NA | 4.39 | 1.01 | 1.18 | 2.98 | 81.6 |
| 5 | 44 | F | 45 | 3.15 | NA | 5.86 | NA | 3.72 | 1.25 | 1.49 | 1.73 | 117.02 |
| 6 | 45 | F | 45 | 4.34 | NA | 4.88 | NA | 3.68 | 0.46 | 1.42 | 1.85 | 116.2 |
| 7 | 27 | M | 81 | 3.30 | NA | 4.67 | NA | 3.70 | 0.68 | 1.39 | 2.28 | 114.14 |
| 8 | 37 | F | 50 | 3.38 | NA | 4.47 | NA | NA | NA | NA | NA | 118.73 |
| 9 | 59 | M | 84 | 4.9 | NA | 4.76 | NA | 4.52 | 1.31 | 1.24 | 2.86 | 87.24 |
| 10 | 45 | F | 73 | 6.1 | NA | 5.07 | NA | 3.3 | 0.52 | 1.32 | 1.65 | 85.69 |
| 11 | 41 | M | 79 | 4.48 | NA | 5.21 | NA | 4.47 | 1.61 | 1.35 | 2.68 | 106.02 |
| 12 | 23 | F | 52 | 3.04 | NA | 5.22 | NA | NA | NA | NA | NA | 129.32 |
| 13 | 31 | M | 85 | 4.38 | NA | 4.60 | NA | 4.71 | 1.46 | 1.19 | 3.07 | 104.69 |
| 14 | 36 | M | 96 | 5.01 | NA | 4.30 | NA | 4.37 | 0.97 | 1.34 | 2.46 | 87.25 |
| 15 | 42 | F | 57 | 4.43 | NA | 4.35 | NA | 4.8 | 1.1 | 1.41 | 2.85 | 109.79 |
| mean ± SD | 43.80 ± |  | 69.67 ± 17.07 | 4.54 ± 1.28 |  | 4.92 ± 0.43 |  | 4.28 ± 0.54 | 1.04 ± 0.10 | 1.35 ± 0.10 | 2.61 ± 0.59 | 102.33 ± 14.83 |
|  | 14.29 |  |  |  |  |  |  |  |  |  |  |  |
| DKD group (n = 22) | | | | | | | | | | | | |
| Number | Age (yr) | Sex | SCr  (μmol/L) | BUN  (mmol/ L) | UPR/24h (g) | FBG  (mmol/ L) | HbAlc (%) | CHO  (mmol/ L) | TG  (mmol/L) | HDL-C  (mmol/L) | LDL-C  (mmol/L) | eGFR（ml/min） |
| 1 | 49 | M | 56 | 4.7 | 1.84 | NA | 7.1 | 3.62 | 1.69 | 0.92 | 2.41 | 115.45 |
| 2 | 66 | M | 94 | 8.44 | 1.1 | 4.24 | 7.58 | 4.53 | 2.36 | 1.12 | 2.95 | 72.49 |
| 3 | 63 | F | 36.21 | 5.03 | 1.53 | 17.47 | 9.26 | 6.05 | 3.02 | 2.01 | 3.29 | 109.99 |
| 4 | 61 | M | 105.57 | 8.1 | 4.75 | 4.79 | 6.5 | 4.34 | 2.37 | 1.37 | 2.69 | 65.25 |
| 5 | 52 | F | 44.47 | 3.01 | 2.33 | 4.03 | 5.8 | 6.21 | 1.85 | 1.68 | 3.95 | 111.05 |
| 6 | 67 | M | 81 | 3.8 | 3.7 | 5.01 | 5.91 | 3.35 | 1.59 | 1.16 | 1.66 | 86.18 |
| 7 | 62 | M | 90.11 | 5.92 | 8.61 | 9.21 | 9.8 | 4.23 | 3.79 | 0.95 | 3.05 | 78.47 |
| 8 | 62 | M | 292.61 | 20.01 | 2.27 | 3.46 | 5.6 | 3.9 | 1.1 | 1.19 | 2.58 | 18.89 |
| 9 | 46 | M | 201 | 7.9 | 15.38 | 6.87 | NA | NA | NA | NA | NA | 33.28 |
| 10 | 72 | M | 130 | 8.02 | 2.97 | 4.19 | NA | 3.64 | 3.03 | 1.05 | 2.14 | 46.96 |
| 11 | 51 | M | 77 | 5 | 14.23 | 9.57 | NA | 3.72 | 1.89 | 0.9 | 2.21 | 102.51 |
| 12 | 46 | M | 281.56 | 13.44 | 1.89 | 13.98 | NA | 2.91 | 1.42 | 0.95 | 1.59 | 22.14 |
| 13 | 61 | M | 189.24 | 8.54 | 2.22 | 5.18 | 5.2 | 5.1 | 3.38 | 1.01 | 3.4 | 32.22 |
| 14 | 53 | M | 74.18 | 12.28 | 4.23 | NA | 8.1 | 6.27 | 0.68 | 1.34 | 4.03 | 105.75 |
| 15 | 53 | M | 130.83 | 11.03 | 3.98 | NA | 11.3 | NA | NA | NA | NA | 53.25 |
| 16 | 46 | M | 361.41 | 8.23 | 6.72 | 5.79 | 6.7 | 6.01 | 4.96 | 0.66 | 3.77 | 16.37 |
| 17 | 66 | M | 240 | 25.5 | 5.26 | 11.58 | 7.2 | 3.94 | 1.14 | 1.46 | 2.21 | 23.34 |
| 18 | 42 | F | 525.6 | 16.47 | 8.54 | 6.57 | NA | 10.32 | 14 | 1.12 | 3.97 | 8.07 |
| 19 | 52 | F | 894.5 | 40.97 | 3.86 | 10.76 | 5.4 | 4.46 | 1.6 | 0.99 | 3.02 | 3.95 |
| 20 | 53 | M | 826.87 | 26.51 | 5.57 | NA | NA | 5.09 | 1.41 | 1.83 | 2.93 | 5.73 |
| 21 | 51 | F | 334.32 | 13.72 | 4.51 | 6.18 | 6.1 | 3.93 | 1.8 | 0.98 | 2.65 | 13.09 |
| 22 | 38 | F | 268.36 | 11.74 | 6.63 | NA | 7 | 4.42 | 1.28 | 0.96 | 2.55 | 18.71 |
| mean ± SD | 55.09 ± 9.02 |  | 242.5 ± 235.6 | 12.2 ± 9.11 | 5.10±3.80 | 7.58 ± 3.96 | 7.16 ± 1.72 | 4.80± 1.63 | 2.72 ± 2.85 | 1.18 ± 0.34 | 2.85 ± 0.73 | 51.96 ± 38.51 |
|  |  |  |  |  |  |  |  |  |  |  |  |  |
| MCD group (n = 5) | | | | | | | | | | | | |
| Number | Age (yr) | Sex | SCr  (μmol/L) | BUN  (mmol/ L) | UPR/24h (g) | FBG  (mmol/ L) | HbAlc (%) | CHO  (mmol/ L) | TG  (mmol/L) | HDL-C  (mmol/L) | LDL-C  (mmol/L) | eGFR（ml/min） |
| 1 | 19 | M | 100.36 | 9 | 1.84 | 3.56 | NA | 15.16 | 5.28 | 1.73 | 8.88 | 93.17 |
| 2 | 17 | M | 83.83 | 6.52 | 1.1 | 4.35 | NA | 15.76 | 3.91 | 2.15 | 10.21 | 117.46 |
| 3 | 50 | F | 52.27 | 2.67 | 1.53 | 4.25 | NA | 9.09 | 2.64 | 1.87 | 5.98 | 107.13 |
| 4 | 20 | F | 53.12 | 4.66 | 4.75 | 4.89 | NA | 10.13 | 5.19 | 0.97 | 5.99 | 131.57 |
| 5 | 70 | F | 86.52 | 10.05 | 2.33 | 3.92 | NA | 9.64 | 3.59 | 1.22 | 6.46 | 58.72 |
| mean ± SD | 35.2 ± 23.74 |  | 75.22 ± 21.5 | 6.58 ± 3.04 | 2.31±1.44 | 4.19 ± 0.50 |  | 11.96± 3.23 | 4.12 ± 1.12 | 1.59 ± 0.48 | 7.50± 1.93 | 103.72 ± 24.86 |
|  |  |  |  |  |  |  |  |  |  |  |  |  |
| SGN group (n = 6) | | | | | | | | | | | | |
| Number | Age (yr) | Sex | SCr  (μmol/L) | BUN  (mmol/ L) | UPR/24h (g) | FBG  (mmol/ L) | HbAlc (%) | CHO  (mmol/ L) | TG  (mmol/L) | HDL-C  (mmol/L) | LDL-C  (mmol/L) | eGFR（ml/min） |
| 1 | 31 | F | 287.94 | 11.66 | 0.8 | 3.49 | NA | 5.53 | 3.96 | 1.1 | 3.81 | 18.05 |
| 2 | 37 | M | 261.53 | 9.72 | 2.59 | 7.16 | NA | 4.78 | 0.9 | 1.68 | 2.49 | 25.79 |
| 3 | 69 | F | 247 | 3.2 | NA | 5.18 | NA | NA | NA | NA | NA | 16.63 |
| 4 | 39 | F | 327 | 13.07 | 0.82 | 5.38 | NA | 3.12 | 1.84 | 0.88 | 1.54 | 14.63 |
| 5 | 45 | F | 216.96 | 10.42 | 0.18 | 4.29 | NA | 5.74 | 1.06 | 2.31 | 3.01 | 23.03 |
| 6 | 64 | M | 140 | 7.83 | 0.68 | 5.57 | NA | 4.6 | 2.26 | 0.92 | 2.65 | 45.41 |
| mean ± SD | 47.5 ±  15.46 |  | 246.7 ± 64.25 | 9.32 ± 3.48 | 1.01±0.912 | 5.18 ± 1.25 |  | 4.75± 1.03 | 2.00 ± 1.23 | 1.38 ± 0.61 | 2.7± 0.82 | 23.92 ± 10.94 |

FBG, Fasting blood-glucose; SCr, Serum creatinine; BUN, Blood urea nitrogen; CHO, Cholesterol; TG, Triglyceride; LDL-C, Low-density lipoprotein Cholesterol; HDL-C, High-density lipoprotein Cholesterol; eGFR, Estimated glomerular filtration rate; HbA1c,Hemoglobin Alc; UPR, [urine](javascript:;) [protein](javascript:;) [quantitation](javascript:;); NA, Not available; F, Female; M, Male; DKD, Diabetic kidney disease; MCD, minimal change disease; SGN, Sclerosing glomerulonephritis.

Data are expressed as mean ± SD.

**Supplementary Table 3. Physicochemical parameters of** ***db/db* mice. Related to Figure 1.**

| **Variable** | ***db/db* diabetic mice** | | | |
| --- | --- | --- | --- | --- |
|  | **16w** | | **40w** | |
|  | **WT** | ***db/db*** | **WT** | ***db/db*** |
| BW (g) | 24.33 ± 1.56 | 52.98 ± 2.27* | 24.45 ± 2.58 | 43.09 ± 13.91*^#^ |
| KW (g) | 0.39 ±0.03 | 0.51 ±0.03* | 0.37 ±0.09 | 0.43 ±0.11*# |
| FBG (mmol/L) | 4.72 ±0.58 | 26.84 ±2.94* | 5.27 ±1.23 | 19.15 ±7.54*^#^ |
| SCr (umol/L) | 31.56 ±9.29 | 25.33 ±5.92 | 21.11 ±4.89 | 33.96 ±3.31*^#^ |
| TG (mmol/L) | 1.63 ±0.55 | 3.38 ±1.40* | 0.98 ±0.22 | 1.41 ±0.47*^#^ |
| UA (ng/ml) | 6.43 ±0.58 | 7.68 ±0.17* | 10.39 ±0.74 | 12.01 ±0.22*^#^ |

BW, Body weight; KW, Renal weight; FBG, Fasting blood-glucose; SCr, Serum creatinine; TG, Triglyceride; UA, urinary albumin. Data are expressed as mean ± SD. **P* < 0.05 vs. WT mice, ^#^*P* < 0.05 vs. 16-week-old WT male mice (n=5); 16-week-old *db/db* male mice (n=6); 40-week-old WT male mice (n=10); 40-week-old *db/db* male mice (n=10).

**Supplementary Table 4. Physicochemical indices of *db/db* mice with knockdown of ENTPD5. Related to Figure 2.**

| **variable** | **Sh-vector** | **Sh-ENTPD5** |
| --- | --- | --- |
| BW (g) | 66.90 ± 3.39 | 57.11 ± 2.26* |
| KW (g) | 0.64 ±0.06 | 0.49 ±0.02* |
| FBG (mmol/L) | 27.43 ±2.89 | 17.60 ±3.50* |
| SCr (umol/L) | 21.18 ±5.61 | 34.18±2.48* |
| TG (mmol/L) | 1.19 ± 0.04 | 1.56 ±0.29* |

BW, Body weight; KW, Renal weight; FBG, Fasting blood-glucose; SCr, Serum creatinine; TG, Triglyceride. Data are expressed as mean± SD. **P* < 0.05(n=6/per group; 12-week-old male mice).

**Supplementary Table 5. Primers for the qRT-PCR.**

| **Gene** | **Forward (5**' **to 3**'**)** | **Reverse (5**' **to 3**'**)** |
| --- | --- | --- |
| *Mus ENTPD5* | TTGGCAGCACTGTCTTCTACA | GCCCGCATCAAACATAATTCC |
| *Mus SP1* | TGCAAACCAACAGATCATCCC | TGACAGGTAGCAAGGTGATGT |
| *Gapdh* | TGGTGAAGGTCGGTGTGAAC | GCTCCTGGAAGATGGTGATGG |
| *ENTPD5-PP1* | AGAGGTGGAGAGGAAAAAGG | GGTCCACATGTACATATGTGTGA |
| *ENTPD5-PP2* | CACATATAAGTATGTACATTGG | TTGGCACCAAACTTGAGATGT |
| *ENTPD5-PP3* | ACTTTATACATCCTCTACACG | AGAGTCCCCTCTCCCTGGGCCT |

**Supplementary Table 6. Key resources table**

**Reagent or resource Source Identifier**

**Antibodies**

| Anti-ENTPD5 antibody (1:500) | | Santa Cruz biotechnology | Cat# sc-377172 |
| --- | --- | --- | --- |
| Anti-ENTPD5 antibody (1:2000) | | Abcam | Cat# ab92542 |
| Anti-Cleaved Caspase-3 antibody (1:1000) | | Cell signaling technology | Cat# 9664S |
| Anti-SP1 antibody (1:1000) | | Abcam | Cat# ab227383 |
| Anti-Caspase-12 antibody (1:1000) | | ABclonal Technology | Cat# A0217 |
| Anti-Phospho-PERK antibody (1:1000) | | Affifinity Biosciences | Cat# DF7576 |
| Anti-Phospho-IRE1 antibody (1:1000) | | Affifinity Biosciences | Cat# AF7150 |
| ATF6 Monoclonal antibody (1:1000) | | Proteintech | Cat# 66563-1-AP |
| Anti-BCL-2 antibody (1:1000) | | Cell signaling technology | Cat# 3498T |
| Anti-Collagen Type III antibody (1:1000) | | Proteintech | Cat# 22734-1-AP |
| Anti-CHOP (L63F7) antibody (1:1000) | | Cell signaling technology | Cat# 2895S |
| Anti-DR5 antibody (1:1000) | | Baijia Biotechnology | Cat# IPB10596 |
| Anti-Bax antibody (1:1000) | | Cell signaling technology | Cat# 2772S |
| Anti-EGFR antibody (1:1000) | | Abcam | Cat# ab52894 |
| Anti-O-GlcNAc antibody (1:1000) | | Santa Cruz biotechnology | Cat# sc-59623 |
| Anti-GFAT Polyclonal antibody (1:1000) | | Proteintech | Cat# 14132-1-AP |
| Anti-Fibronectin antibody (1:1000) | | Santa Cruz biotechnology | Cat# sc-59826 |
| Anti-E-cadherin antibody (1:1000) | | Proteintech | Cat# 20874-1-AP |
| Anti-Vimentin antibody (1:1000) | | Proteintech | Cat# 10366-1-AP |
| Anti-GAPDH antibody (1:1000) | | Xianzhi Biotechnology | Cat# AB-P-R 001 |
| Anti-alpha Tubulin antibody (1:10000) | | Abcam | Cat# ab7291 |
| **Chemicals and Peptides** | | | |
| Palmitic acid | | Sigma-Aldrich | Cat# P0500 |
| D-glucose | | Solarbio | Cat# G8150 |
| Proteases, phosphatase inhibitors | | Kangcheng Biotechnology | Cat# KC-440 |
| Lipofectamine 2000 Transfection Reagent | | Invitrogen | Cat# 11668019 |
| TRIzol Reagent | | invitrogen | Cat# 15596026-100ML |
| Lipofectamine RNAiMAX Transfection Reagent | | ThermoFisher | Cat# 13778030 |
| Con A | | Sigma Aldrich | Cat#11028-71-0 |
| UDP-GlcNAc | | Sigma-Aldrich | Cat# U4375 |
| **The important kit** | | | |
| FITC Annexin V Apoptosis Detection Kit I | Becton,Dickinson and Company | | Cat# 556547 |
| TUNEL Cell apoptosis Detection Kit | Kgi Biotechnology | | Cat# KGA703 |
| BCA protein concentration determination kit at 500T | Beyotime Biotechnology | | P001Cat# 2 |
| Triglyceride (TG) assay kit | Jiancheng Bioengineering Research Institute | | Cat# A110-1-1 |
| Urea nitrogen (BUN) test box | Jiancheng Bioengineering Research Institute | | Cat# C013-2-1 |
| Creatinine (Cr) assay kit | Jiancheng Bioengineering Research Institute | | Cat# C011-2-1 |
| FAM-siRNA kit | Sangon Biotech | | N/A |
| Simple ChIP Enzymatic Chromatin IP Kit | Cell signaling technology | | Cat# 9003S |
| Dual-Luciferase Reporter Assay System | Promega | | Cat# E1910 |
| RNA FISH kit | GenePharma | | N/A |
| [Glycogen Periodic Acid Schiff (PAS/Hematoxylin) Stain Kit](https://www.solarbio.com/goods.php?id=1702) | Solarbio | | Cat# G1281 |
| Masson's Trichrome Stain Kit | Solarbio | | Cat# G1340 |
| **Cell lines** | | | |
| Mouse：RTEC | ATCC | | N/A |
| Human: 293T | ATCC | | N/A |
| **Software** | | | |
| ImageJ National Institutes of Health <https://imagej.nih.gov/ij/> | | | |
| GraphPad Prism 8.0 GraphPad <https://www.graphpad.com/> | | | |
| Adobe Photoshop CS6 Adobe <https://www.adobe.com/cn> | | | |
