# Supplementary figures and images for: The UDPase ENTPD5 regulates ER stress-associated renal injury by mediating protein N-glycosylation
Source: Cell Death Dis. 2023 Feb 27;14(2):166. doi: 10.1038/s41419-023-05685-4 (PMC9971188; doi:10.1038/s41419-023-05685-4)

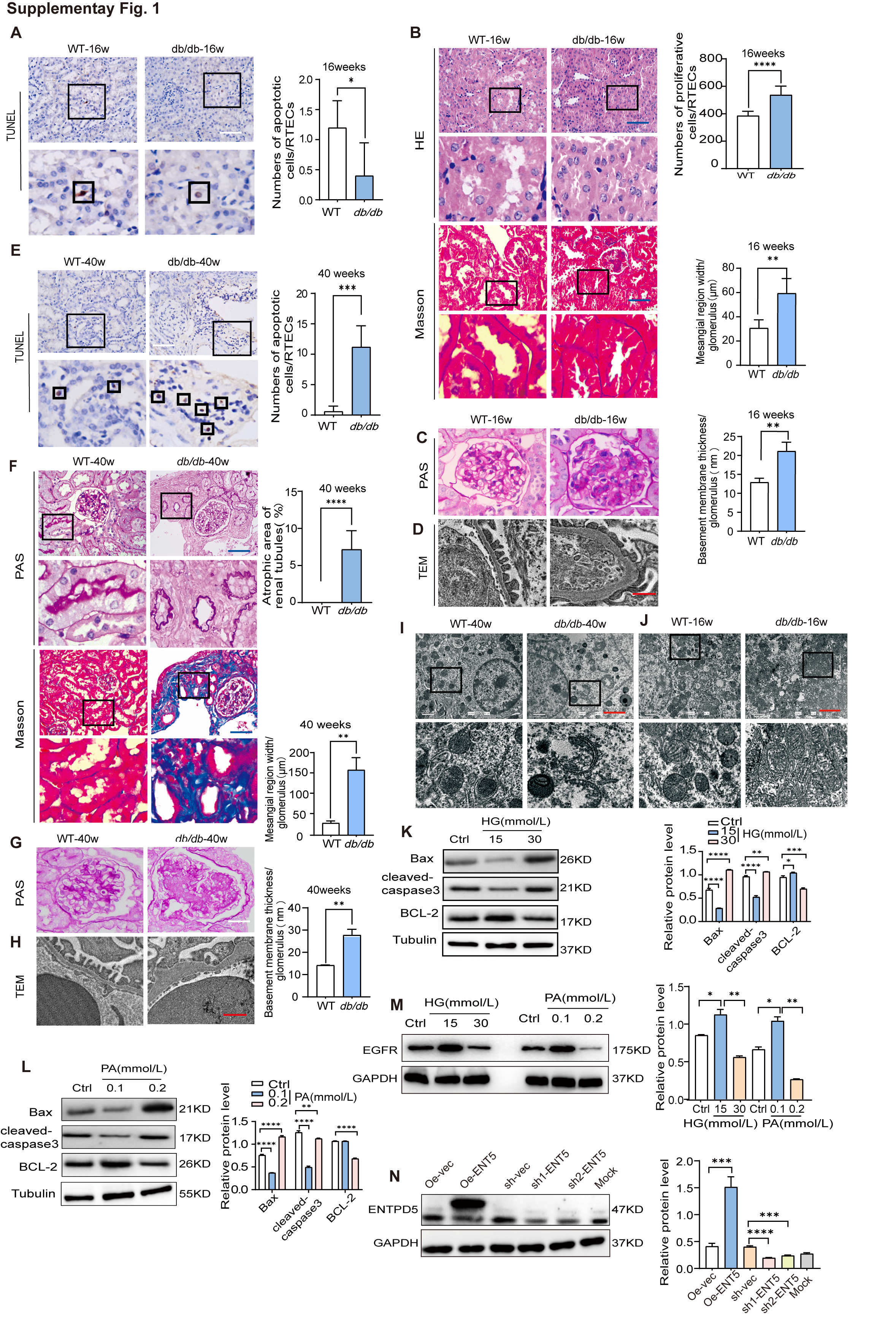

Supplement: Supplementary file 3 — Supplementary Fig.1 [file 41419_2023_5685_MOESM3_ESM.tif]

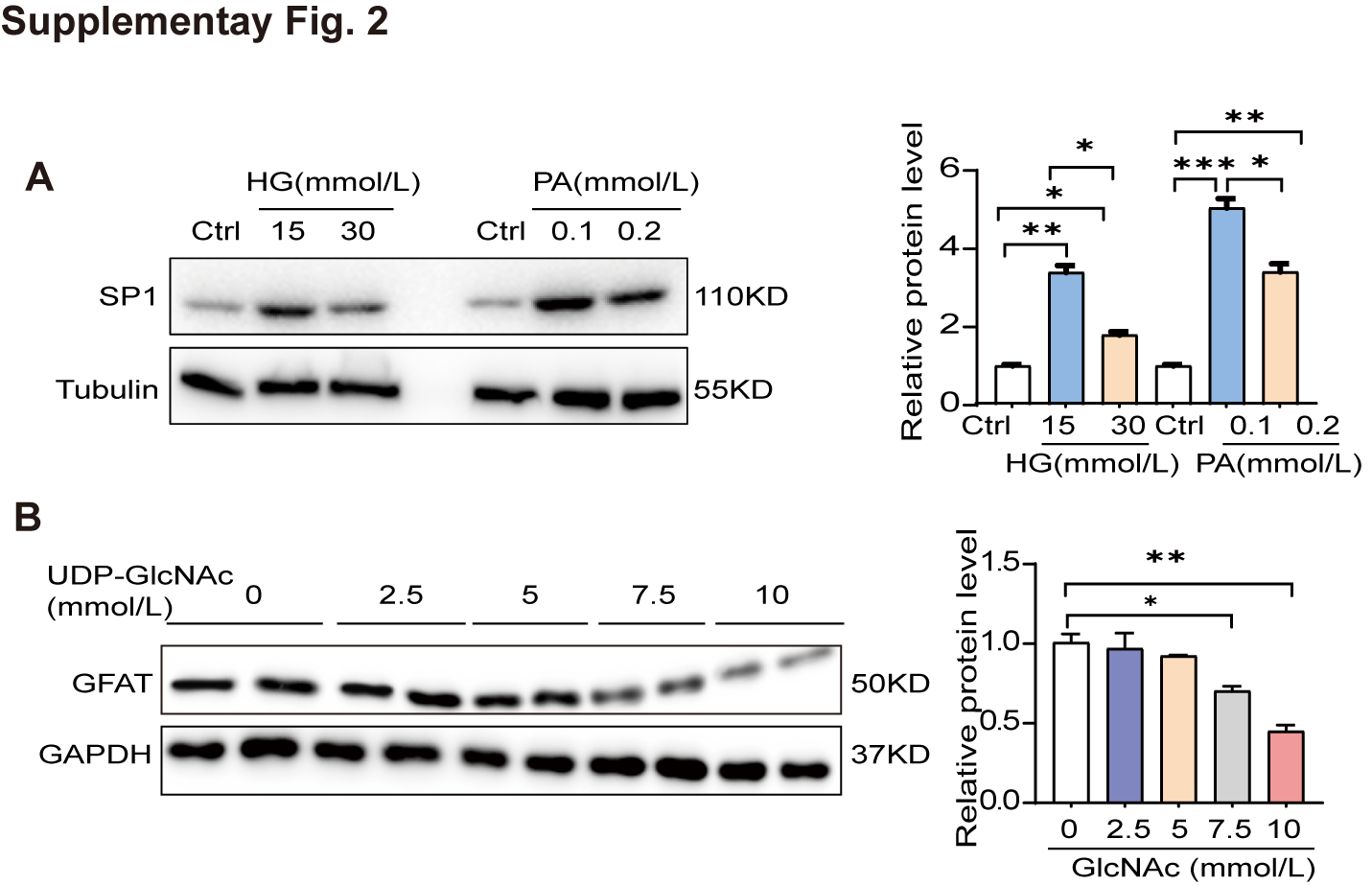

Supplement: Supplementary file 4 — Supplementary Fig.2 [file 41419_2023_5685_MOESM4_ESM.tif]

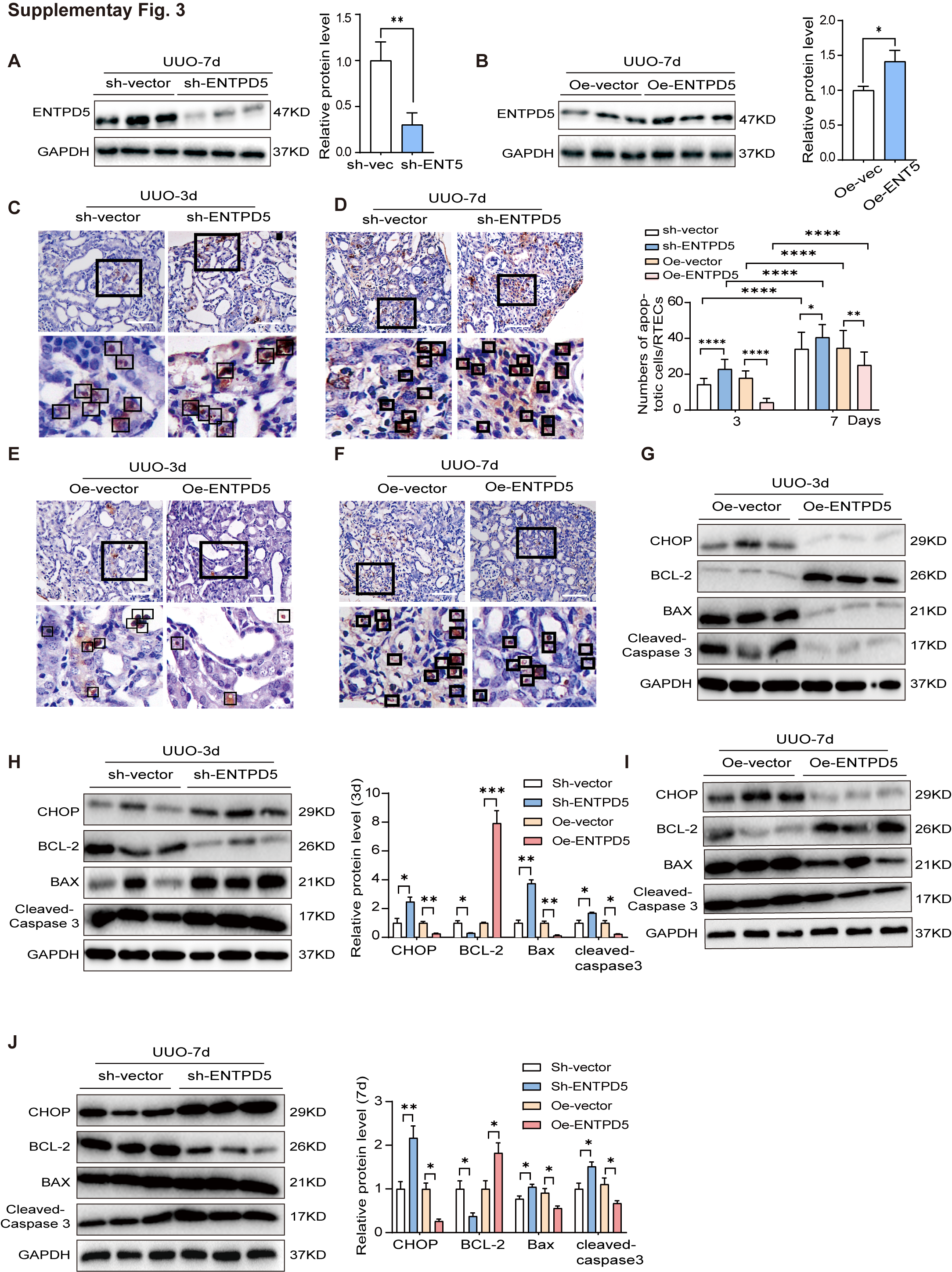

Supplement: Supplementary file 5 — Supplementary Fig.3 [file 41419_2023_5685_MOESM5_ESM.tif]
