# Supplementary material for: The UDPase ENTPD5 regulates ER stress-associated renal injury by mediating protein N-glycosylation
Source: Cell Death Dis. 2023 Feb 27;14(2):166. doi: 10.1038/s41419-023-05685-4 (PMC9971188; doi:10.1038/s41419-023-05685-4)
Supplement: Supplementary file 6 — Supplementary Figure legends [file 41419_2023_5685_MOESM6_ESM.docx]

**Supplementary Figure legends**

**Supplementary Fig. 1** **ENTPD5 drives proliferation or apoptosis in RTECs under diabetic conditions.**

A. Representative TUNEL staining images and quantification of apoptosis rate in 16-week-old *db/db* mice (n=5 per group). Scale bar, red 50μm.

B. Representative HE/Masson staining and proliferation rate of renal tubules in 16-week-old *db/db* mice (n=5 per group), as calculated by HE staining. Scale bar, blue 100μm.

C. Representative PAS staining and mesangial region width of glomerulus in 16-week-old *db/db* mice (n=5 per group), as calculated by PAS staining. Scale bar, white 50μm.

D. Transmission electron microscopy (TEM) images and basement membrane thickness of glomerulus of kidneys in 16-week-old *db/db* mice (n=6 images). Scale bar, red 100nm.

E. Representative TUNEL staining images and quantification of apoptosis rate in 40-week-old *db/db* mice (n=5). Scale bar, red 50μm.

F. Representative PAS/Masson staining and atrophic area of renal tubules in 40-week-old *db/db* mice (n=5), as calculated by PAS staining. Scale bar, blue 100μm.

G. Representative PAS staining and mesangial region width of glomerulus in 40-week-old *db/db* mice (n=5 per group), as calculated by PAS staining. Scale bar, white 50μm.

H. Transmission electron microscopy (TEM) images and basement membrane thickness of glomerulus of kidneys in 40-week-old *db/db* mice (n=6 images). Scale bar, red 100nm.

I-J. Representative TEM images of kidneys in 16-week-old *db/db* mice (J) and 40-week-old *db/db* mice (I) (n=6 images). Scale bar, red 100nm.

K-L. Representative western blot and quantification of apoptosis-related protein expression (BCL-2, Bax and Caspase 3) in RTECs exposed to HG (15 and 30 mmol/L) (K) and PA (0.1 and 0.2 mmol/L) (L) for 48h (n=3 blots).

M. Representative western blot and quantification of EGFR expression in RTECs exposed to HG (15 and 30 mmol/L) or PA (0.1 and 0.2 mmol/L) for 48h (n=3 blots).

N. Representative western blot and quantification of ENTPD5 expression in lentivirus-mediated ENTPD5 knockdown or overexpressing stable RTECs lines (n=3 blots), sh1-ENTPD5 was selected to establish stable cells for subsequent experiments.

Data are mean ± SD. *p < 0.05, **p < 0.01, ***p < 0.001, ****p < 0.0001.

**Supplementary Fig. 2 SP1 regulates the expression of ENTPD5 under diabetic conditions.**

A. Representative western blot and quantification of SP1 expression in RTECs exposed to HG (15 and 30 mmol/L) or PA (0.1 and 0.2 mmol/L) for 48h (n=3 blots).

B. Representative western blot and quantification of GFAT expression in RTECs exposed to NG (5.5 mmol/L) plus different concentrations of UDP-GlcNAc for 48 hours (n=3 blots).

Data are mean ± SD. *p < 0.05, **p < 0.01, ***p < 0.001.

**Supplementary Fig. 3 ENTPD5** [**negative**](C:/Users/Administrator/AppData/Local/youdao/dict/Application/8.9.8.0/resultui/html/index.html#/javascript:;)**ly regulates renal injury in UUO mice.**

A-B. Representative western blot and quantification of ENTPD5 expression in the kidneys of UUO mice with ENTPD5 knockdown (A) or overexpression (B) at 7 days postoperatively (n=3 blots).

C-D. Representative TUNEL staining images and apoptosis rate of renal tubules in UUO mice with ENTPD5 knockdown at 3 days (C) or 7 days (D) postoperatively (n=5 per group). Scale bar, red 50μm.

E-F. Representative TUNEL staining images and apoptosis rate of renal tubules in UUO mice with ENTPD5 overexpression at 3 days (E) or 7 days (F) postoperatively (n=5 per group). Scale bar, red 50μm.

G-H. Representative western blot and quantification of apoptosis-related protein (CHOP, BCL-2, Bax and Caspase 3) in the kidney of UUO mice with ENTPD5 overexpression (G) or knockdown (H) at 3 days postoperatively (n=3 blots).

I-J. Representative western blot and quantification of apoptosis-related protein (CHOP, BCL-2, Bax and Caspase 3) in the kidney of UUO mice with ENTPD5 overexpression (I) or knockdown (J) at 7 days postoperatively (n=3 blots).

Data are mean ± SD. *p < 0.05, **p < 0.01, ***p < 0.001, ****p < 0.0001.
