# Supplementary material for: The UDPase ENTPD5 regulates ER stress-associated renal injury by mediating protein N-glycosylation
Source: Cell Death Dis. 2023 Feb 27;14(2):166. doi: 10.1038/s41419-023-05685-4 (PMC9971188; doi:10.1038/s41419-023-05685-4)

Fig 1J

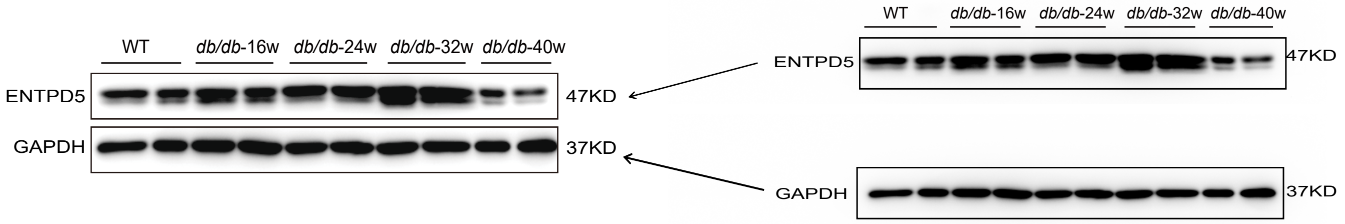

Fig 1K

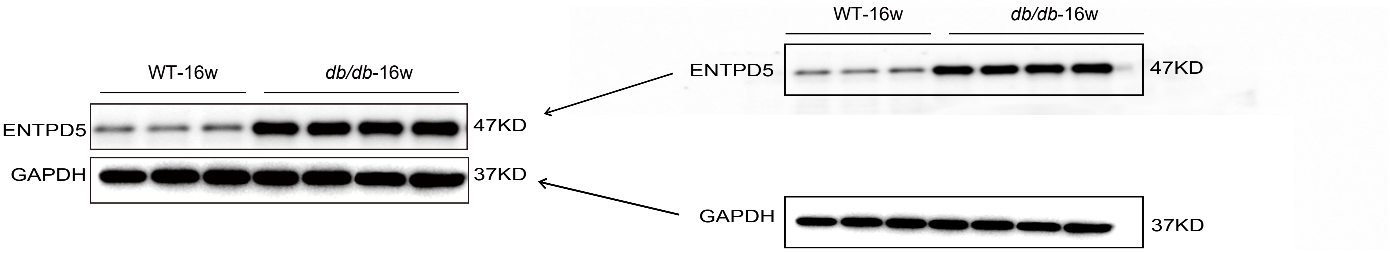

Fig 1M

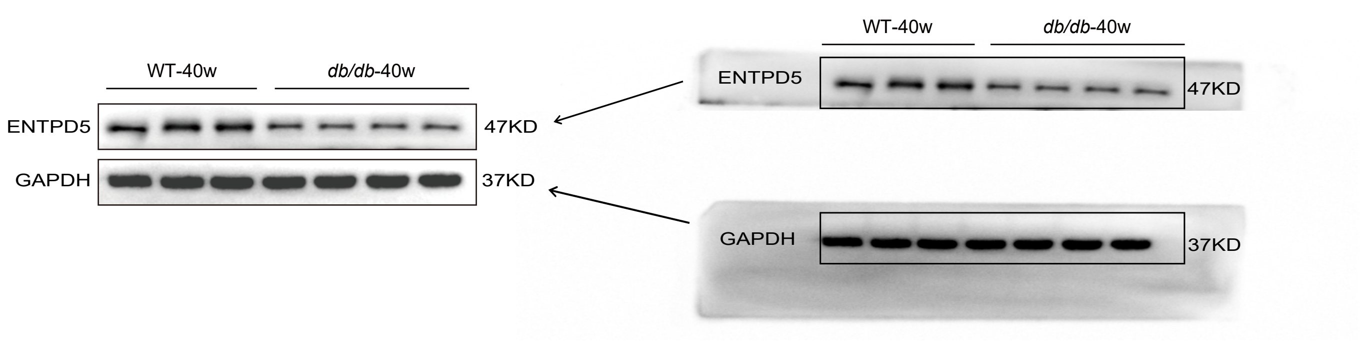

Fig 1R

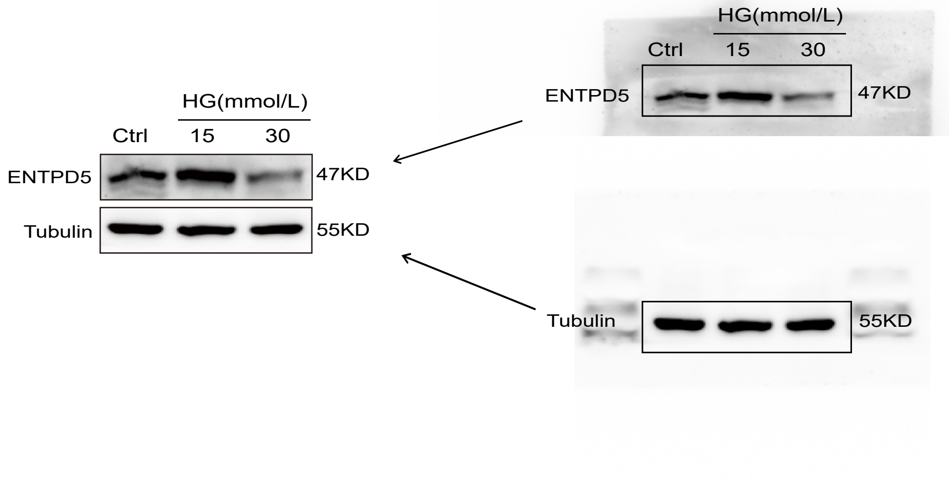

Fig 1S

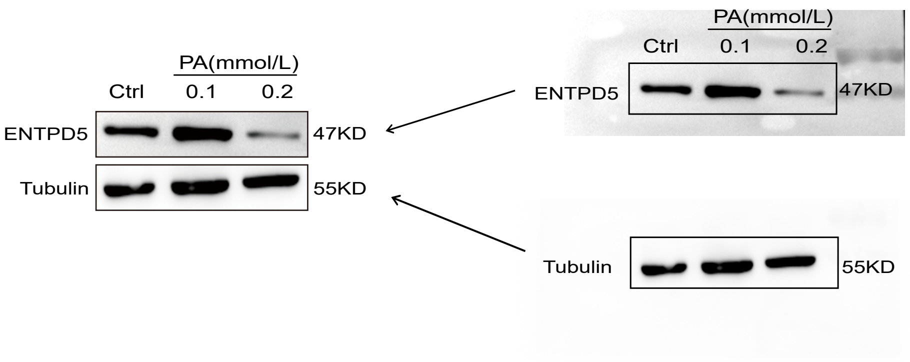

Fig 2D

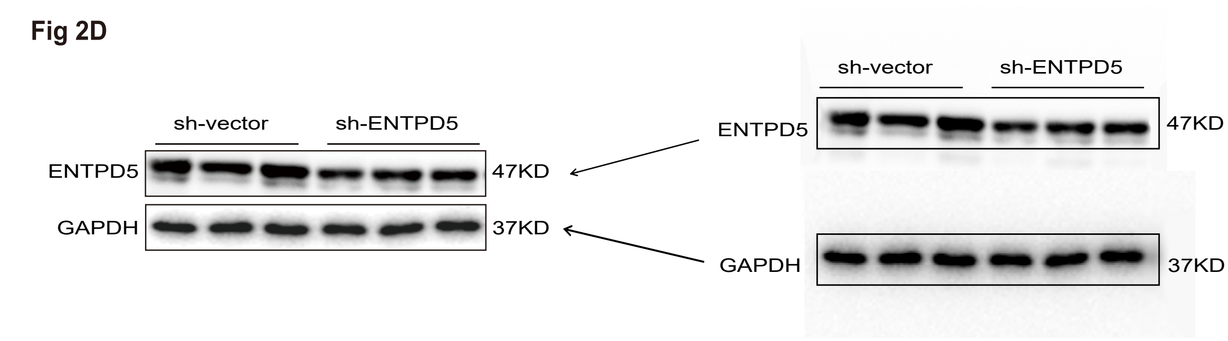

Fig 2L

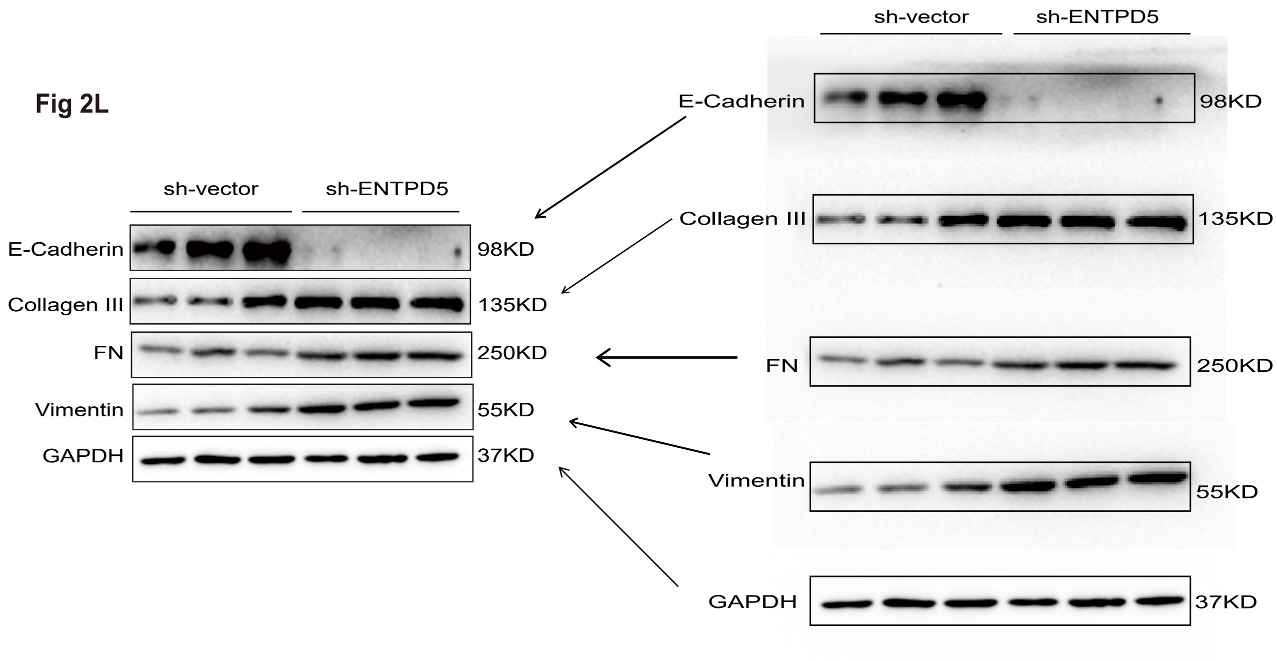

Fig 2O

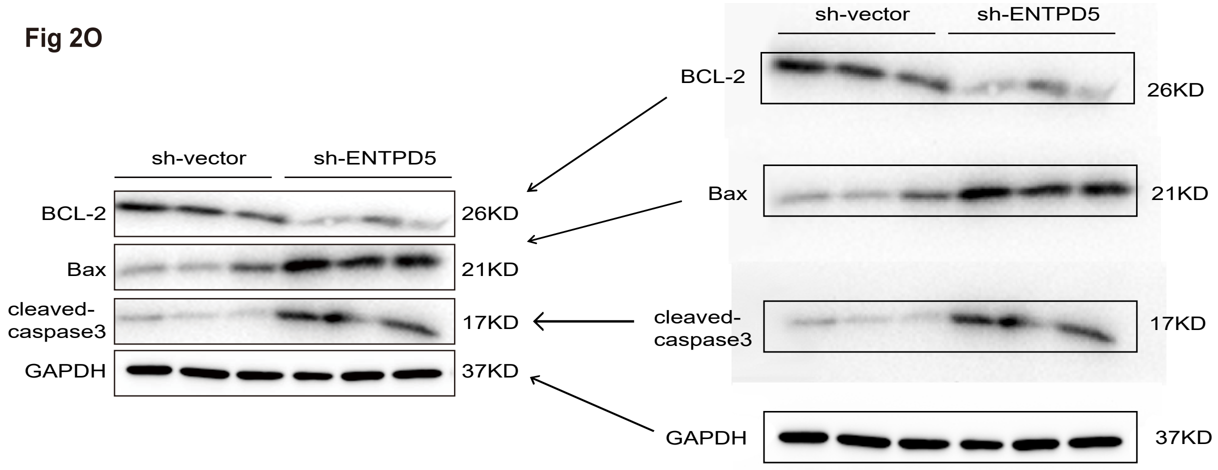

Fig 3B

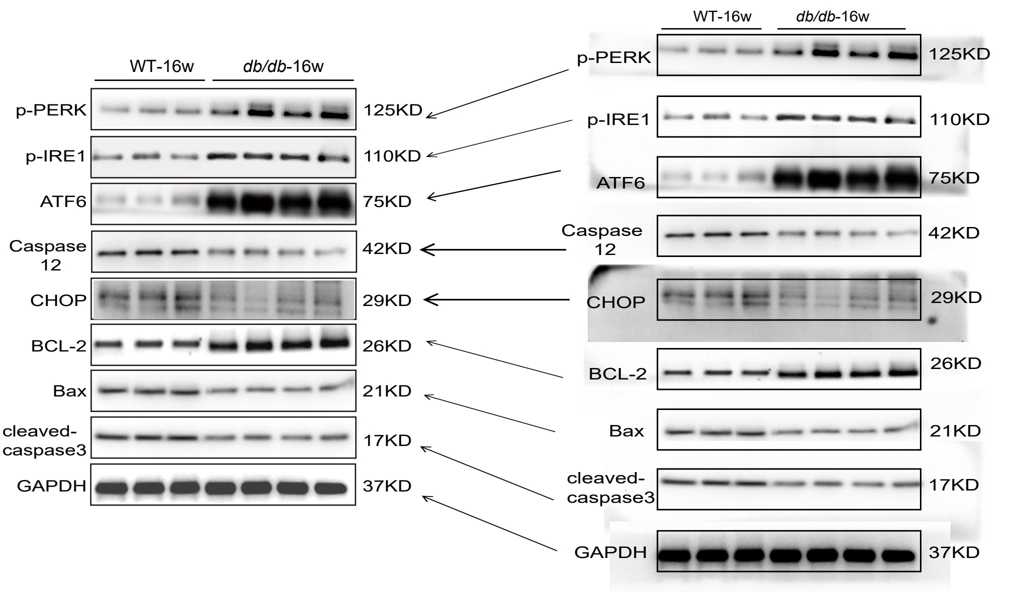

Fig 3C

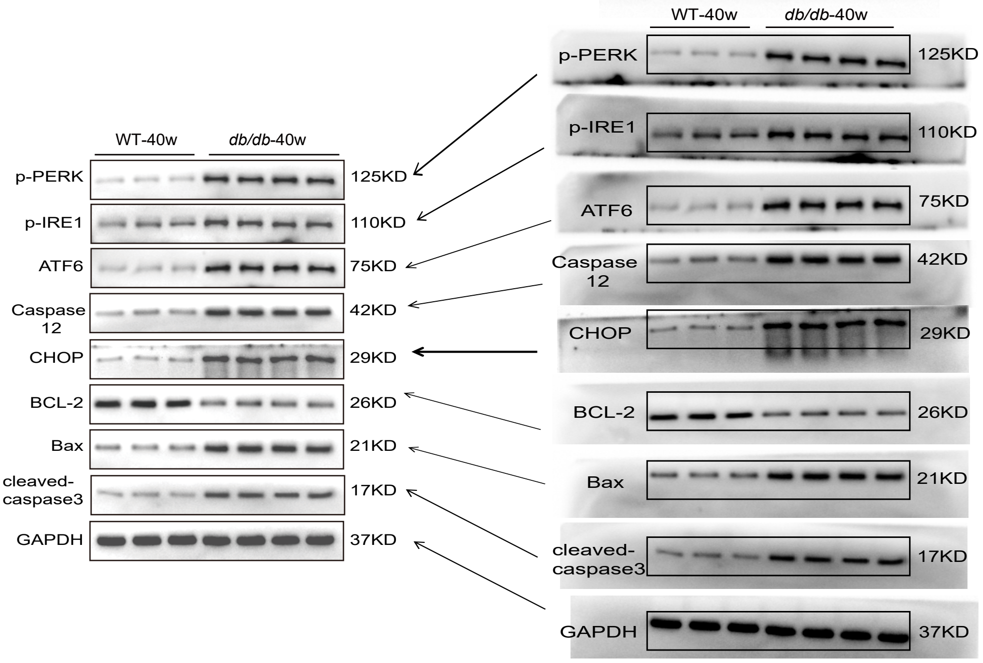

Fig 3D

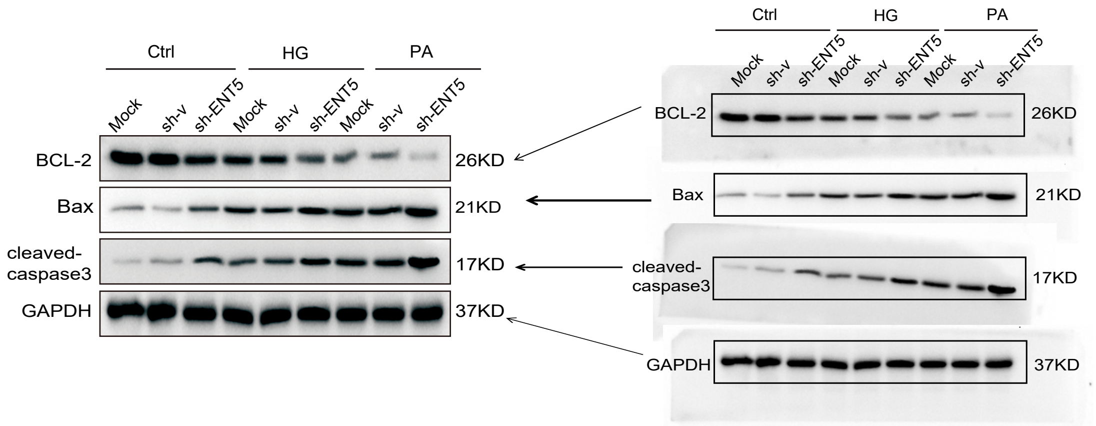

Fig 3E

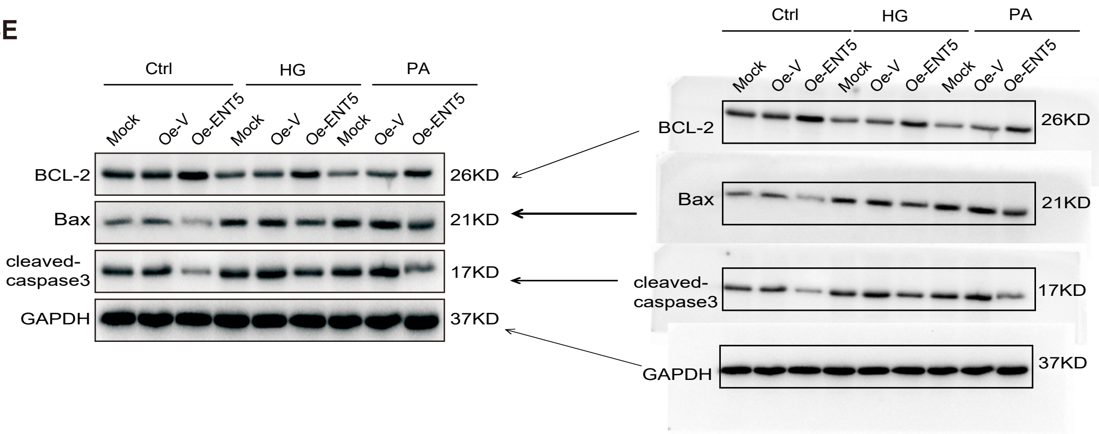

Fig. 4A

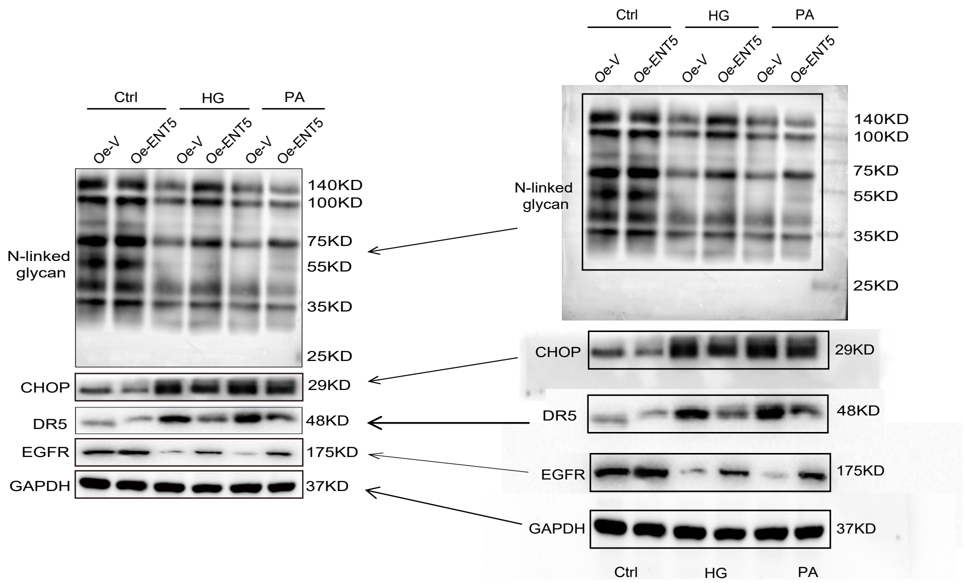

Fig. 4B

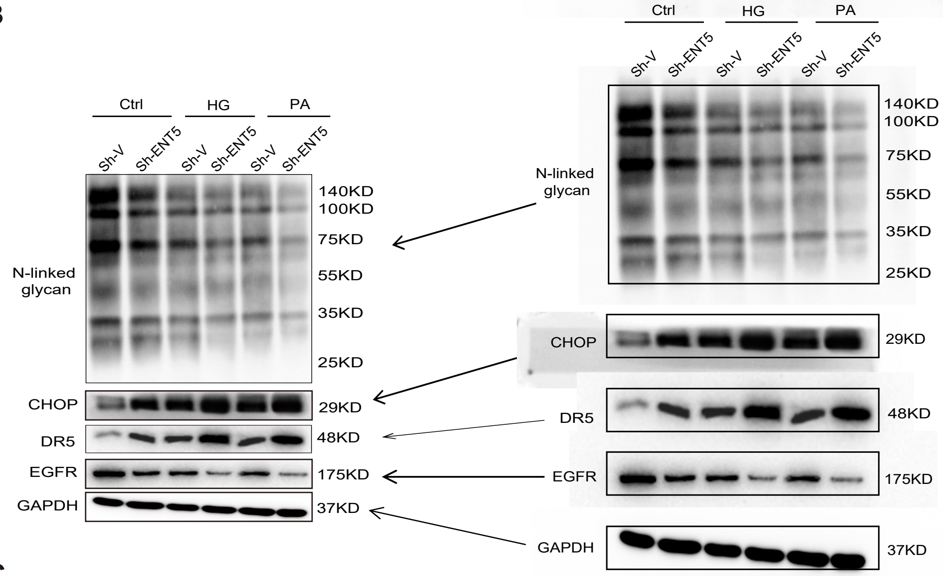

Fig. 4C

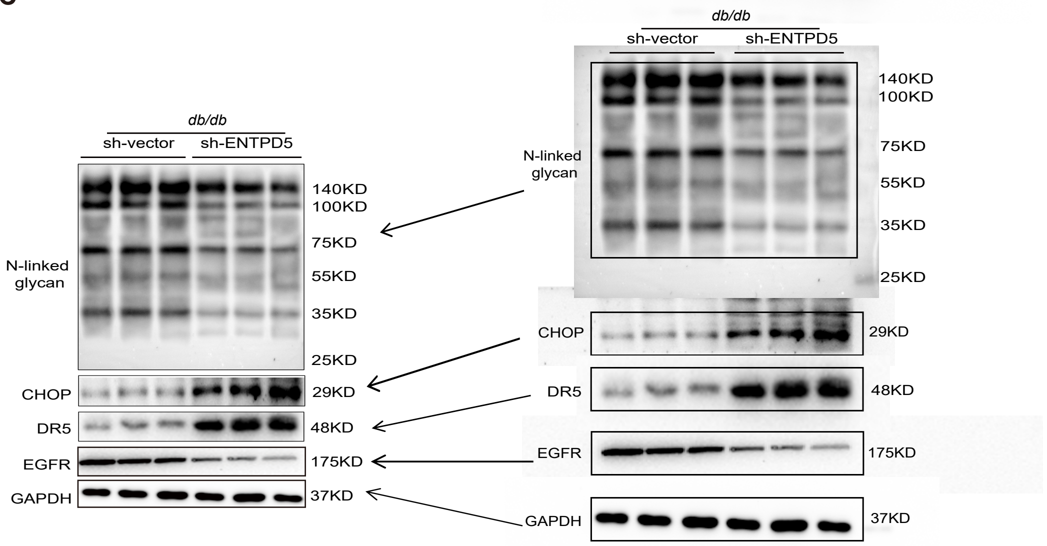

Fig. 4D

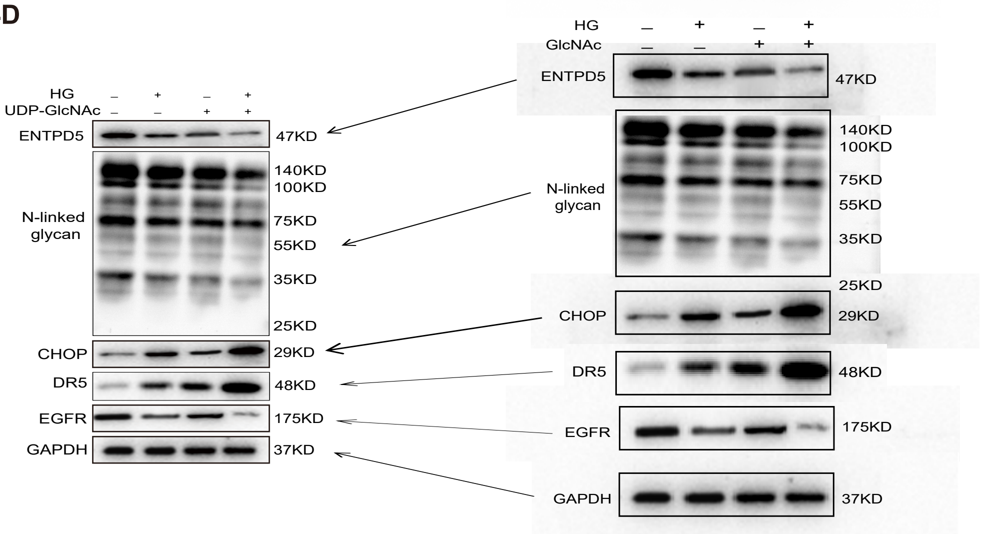

Fig. 5E

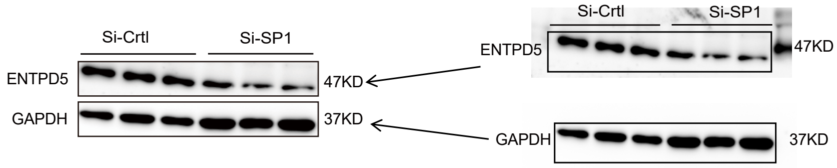

Fig. 5G

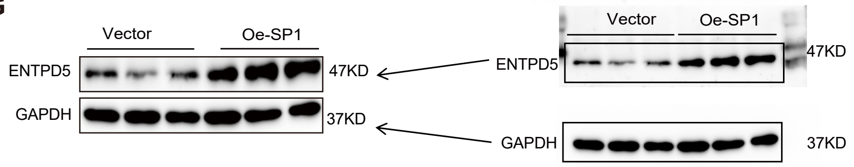

Fig. 5J

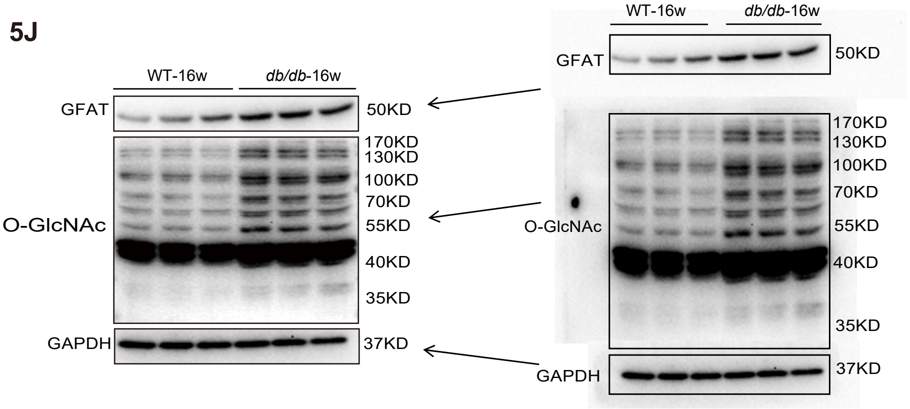

Fig. 5K

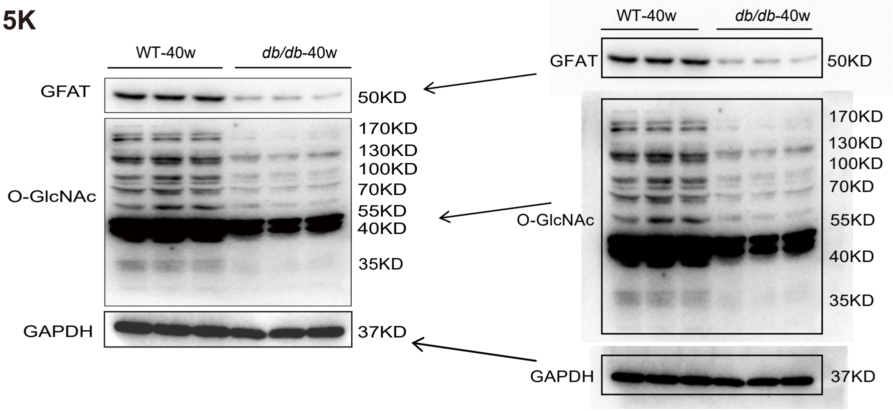

Fig. 5L

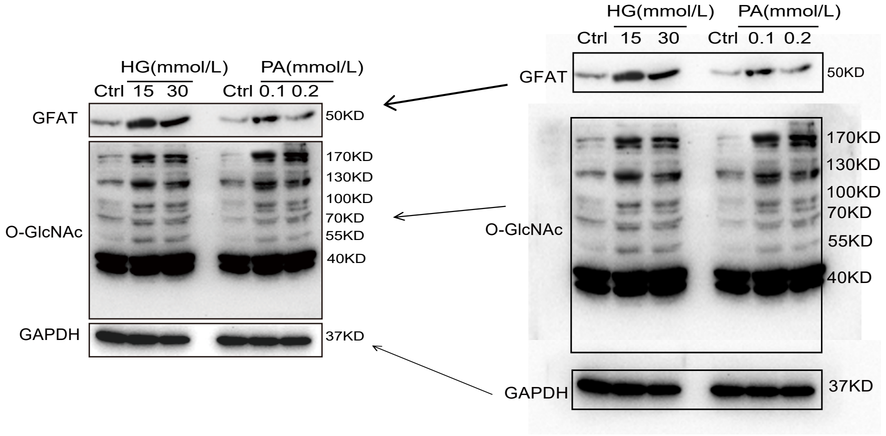

Fig. 5M

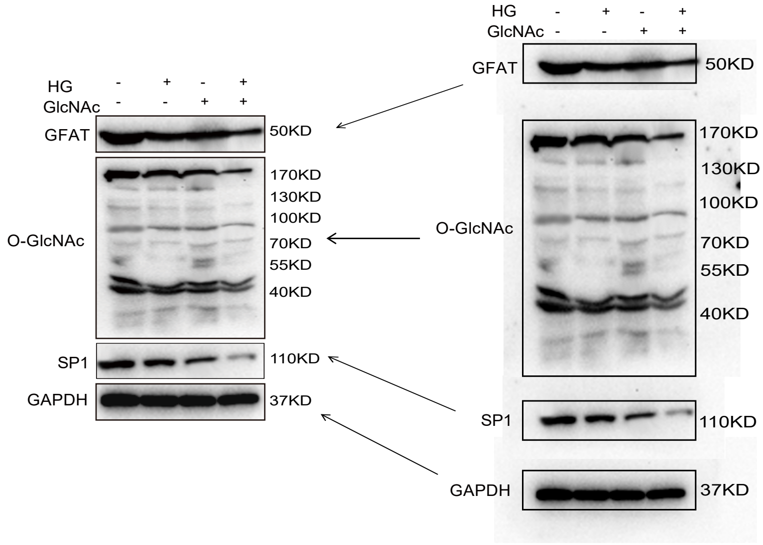

Fig. 6B

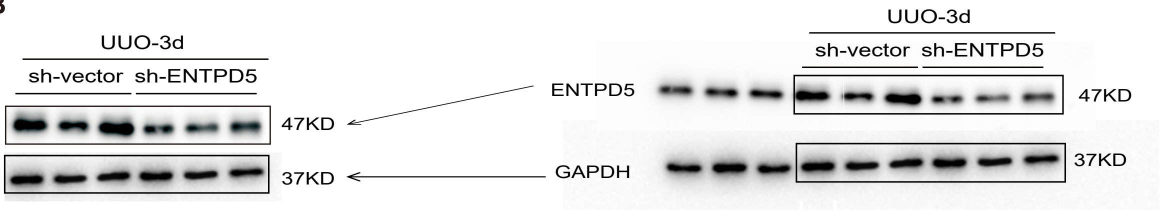

Fig. 6C

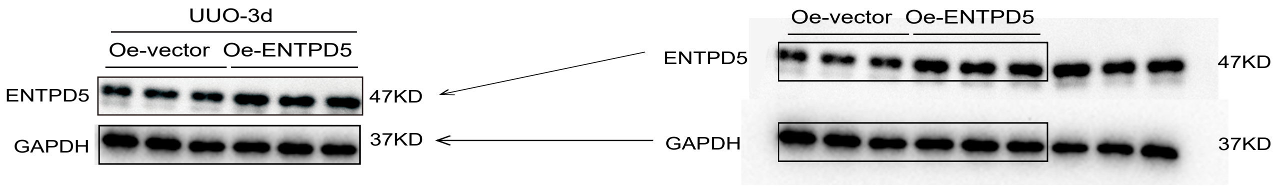

Fig. 6I

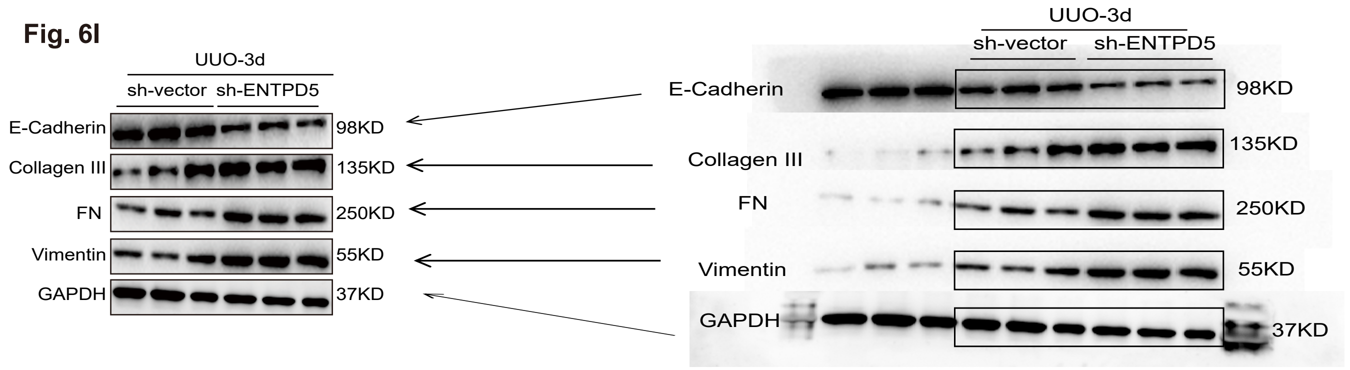

Fig. 6J

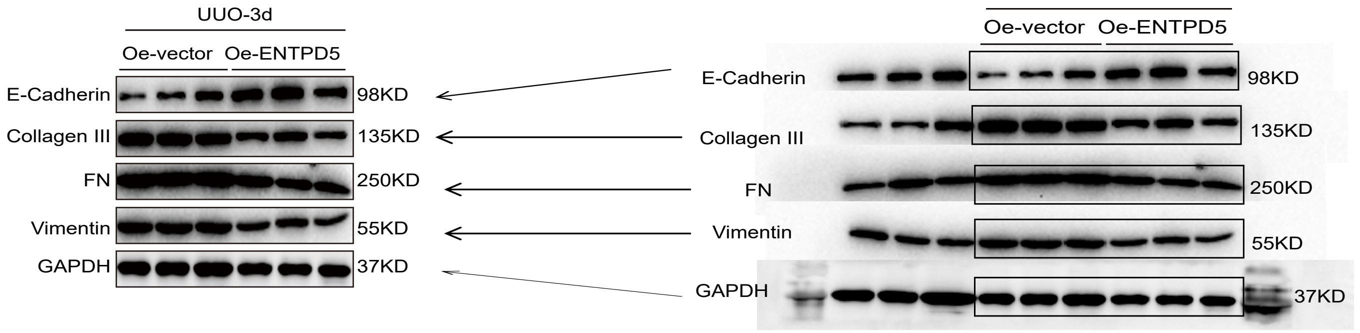

Fig. 6M

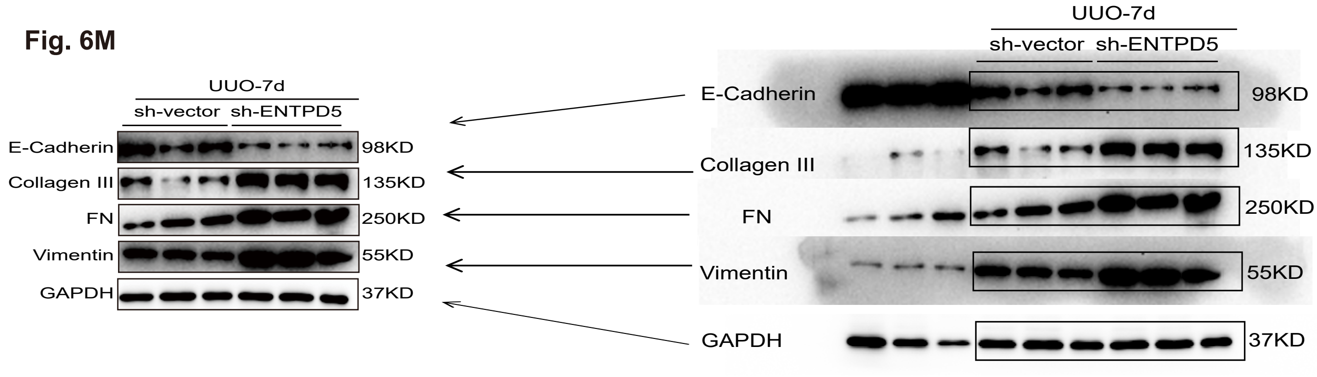

Fig. 6N

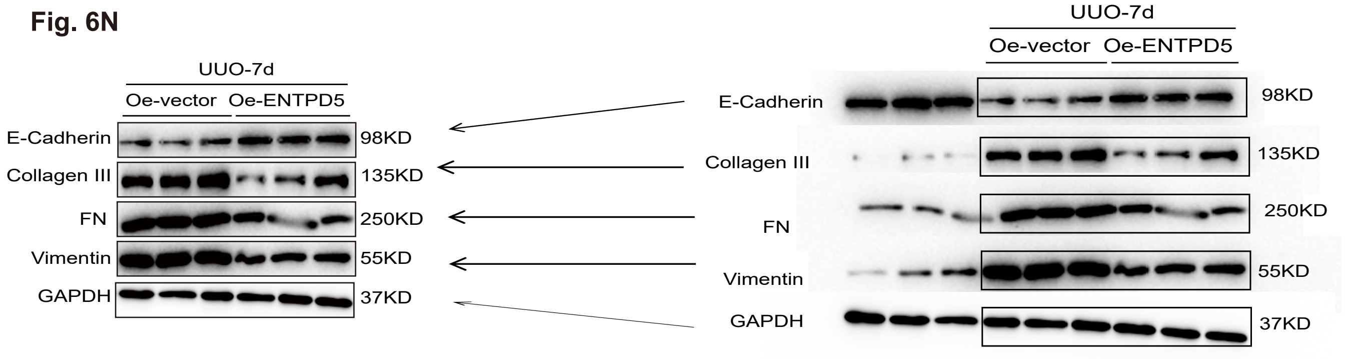

Fig. S1 K

Fig. S1 L

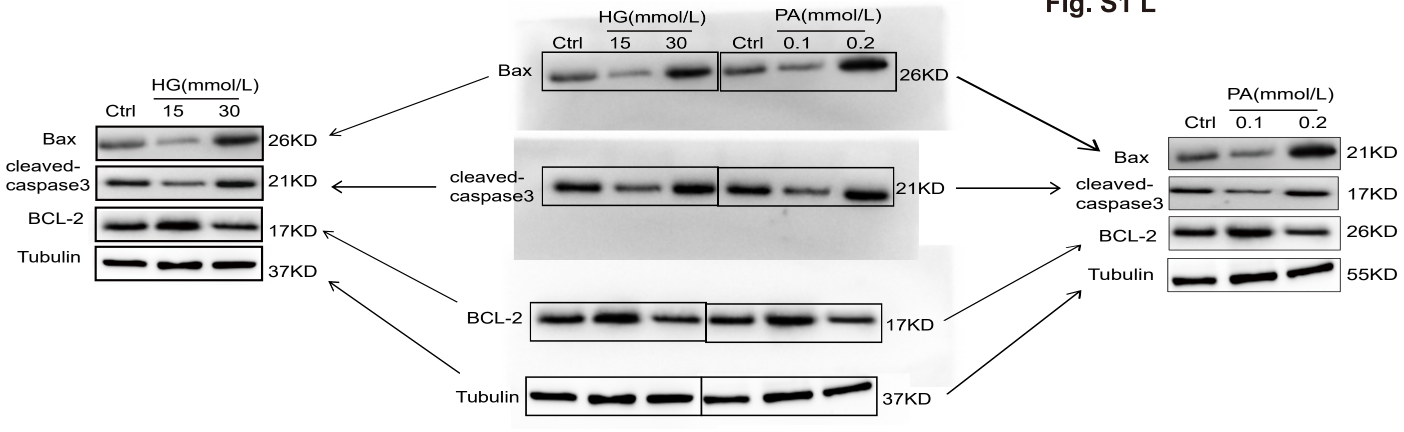

Fig. S1 M

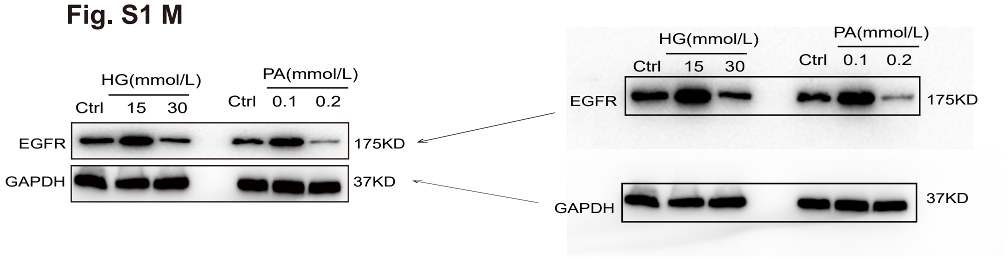

Fig. S1 N

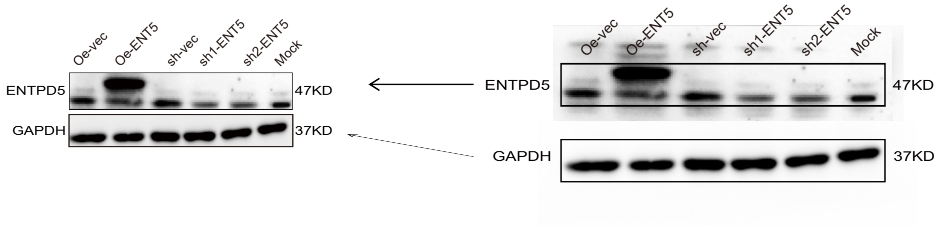

Fig. S2 A

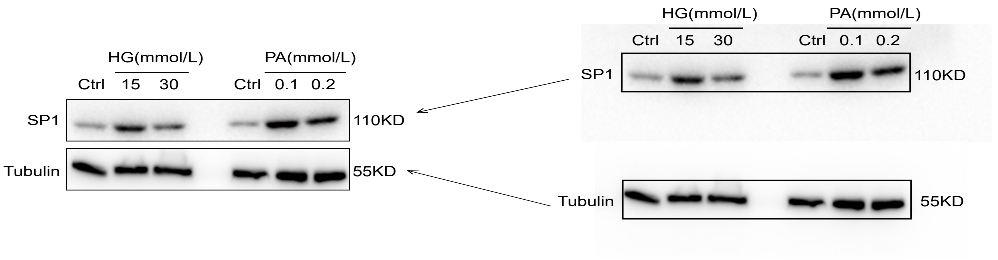

Fig. S2 B

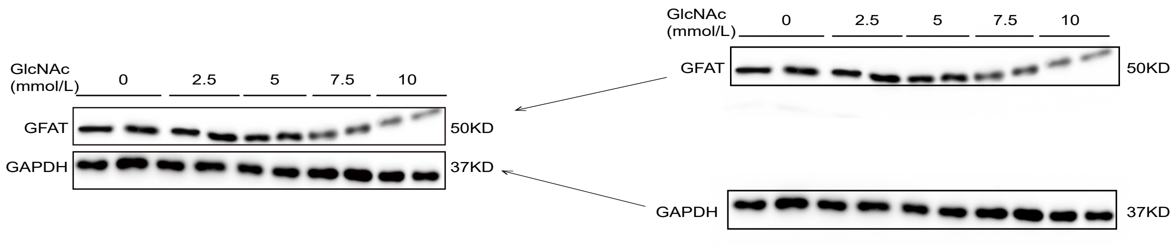

Fig. S3 A

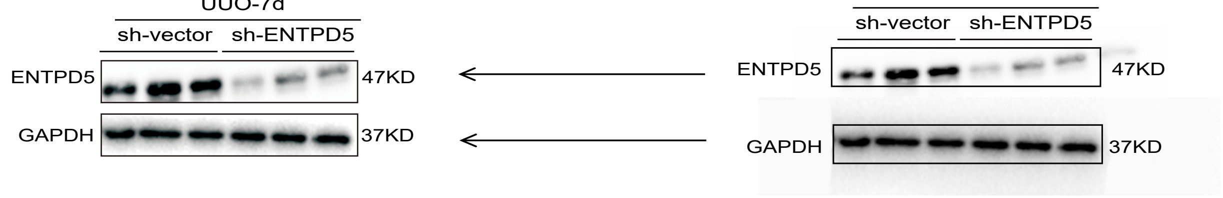

Fig. S3 B

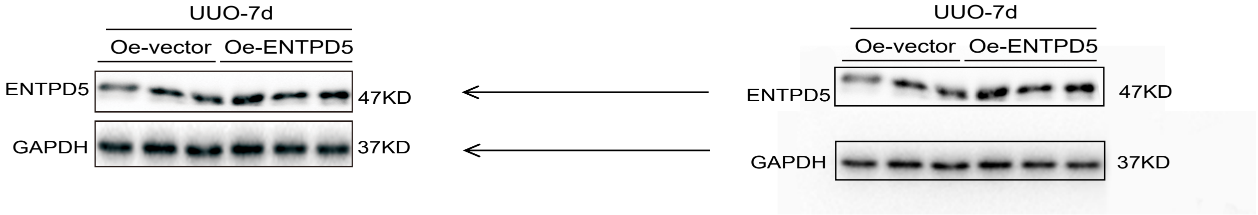

Fig. S3 G

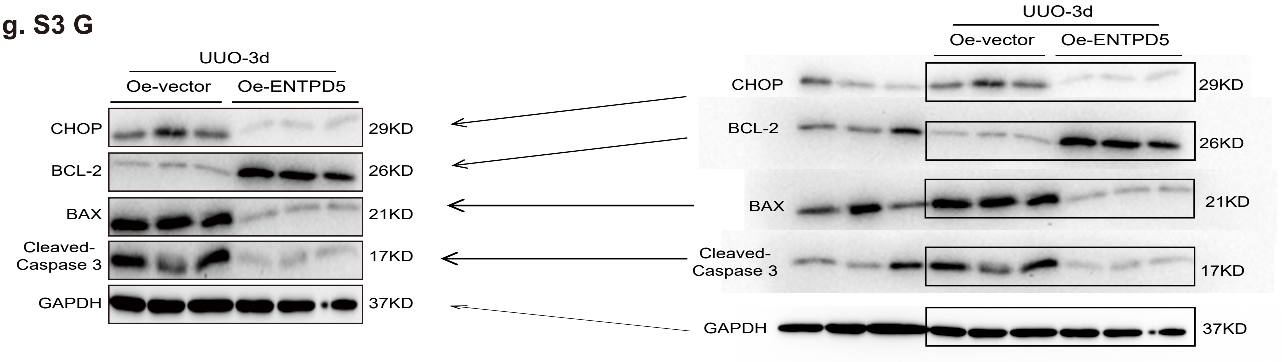

Fig. S3 H

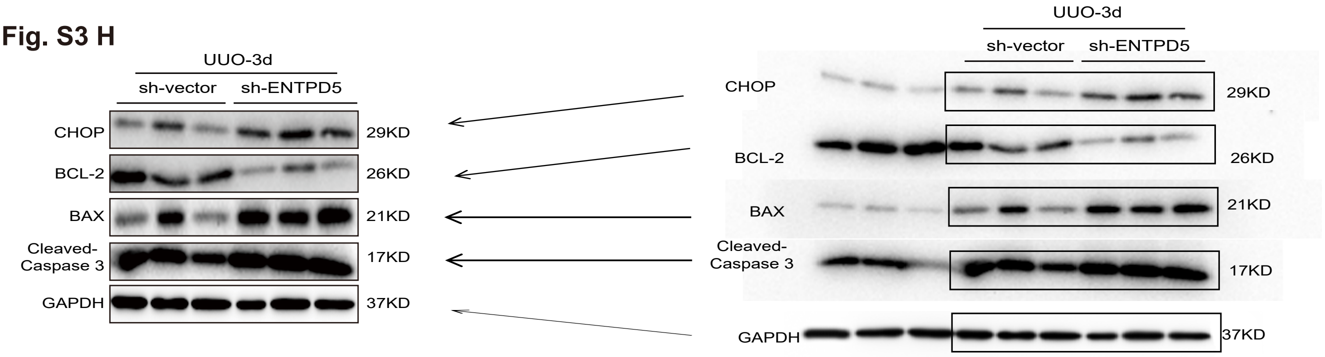

Fig. S3 I

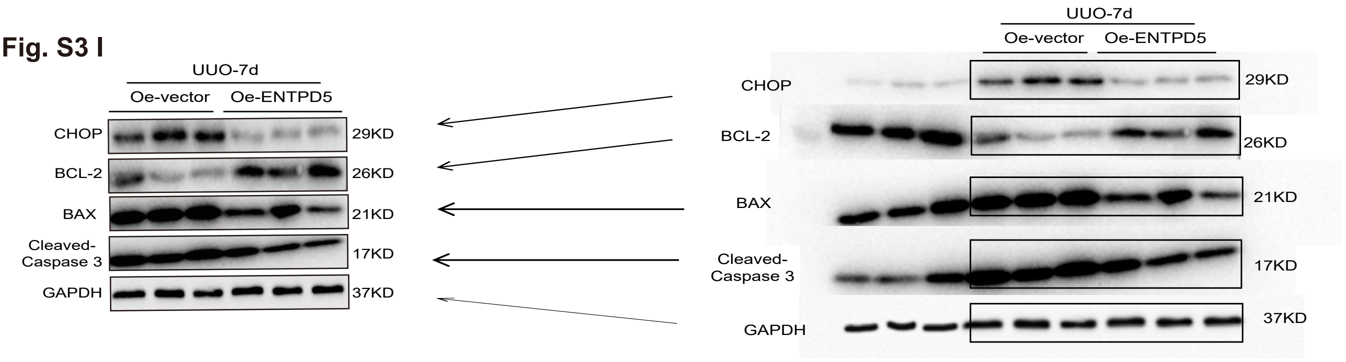

Fig. S3 J

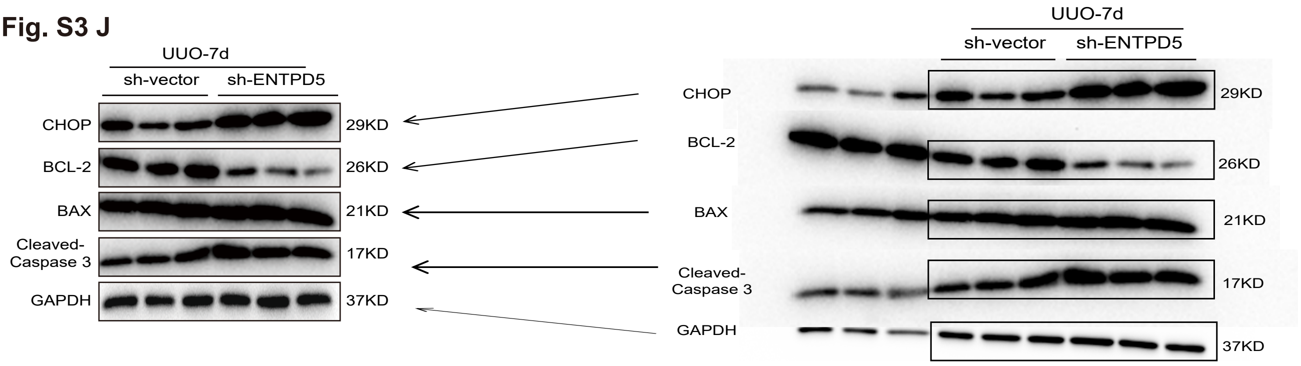

Supplement: Supplementary file 7 — Original Data File [file 41419_2023_5685_MOESM7_ESM.pdf]
